# Supplementary figures and images for: Addressing a critical need: A randomised controlled feasibility trial of acceptance and commitment therapy for bariatric surgery patients at 15–18 months post-surgery
Source: PLoS One. 2023 Apr 25;18(4):e0282849. doi: 10.1371/journal.pone.0282849 (PMC10128967; doi:10.1371/journal.pone.0282849)

**S5 Fig. Individual weight changes**

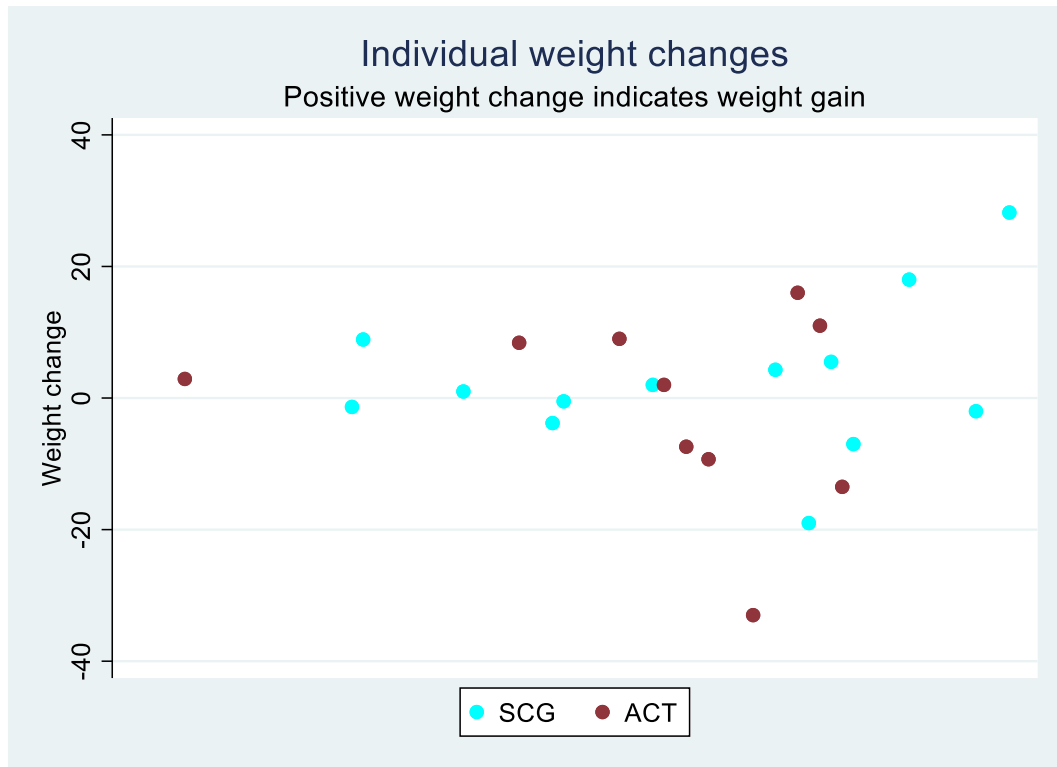

Supplement: S2 Fig — (PDF) [file pone.0282849.s002.pdf]
