# Supplementary material for: Addressing a critical need: A randomised controlled feasibility trial of acceptance and commitment therapy for bariatric surgery patients at 15–18 months post-surgery
Source: PLoS One. 2023 Apr 25;18(4):e0282849. doi: 10.1371/journal.pone.0282849 (PMC10128967; doi:10.1371/journal.pone.0282849)
Supplement: S3 Fig — (PDF) [file pone.0282849.s003.pdf]

S6 Fig. Results from mixed models – unadjusted

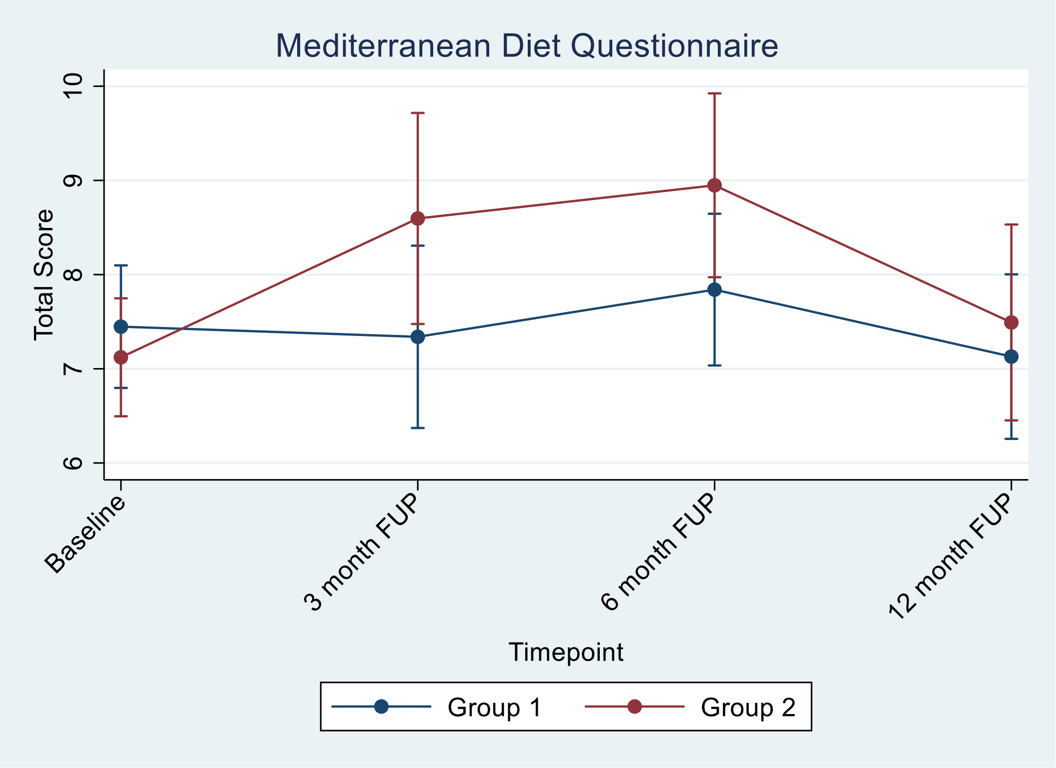

A high score suggests a good adherence to Mediterranean diet principles

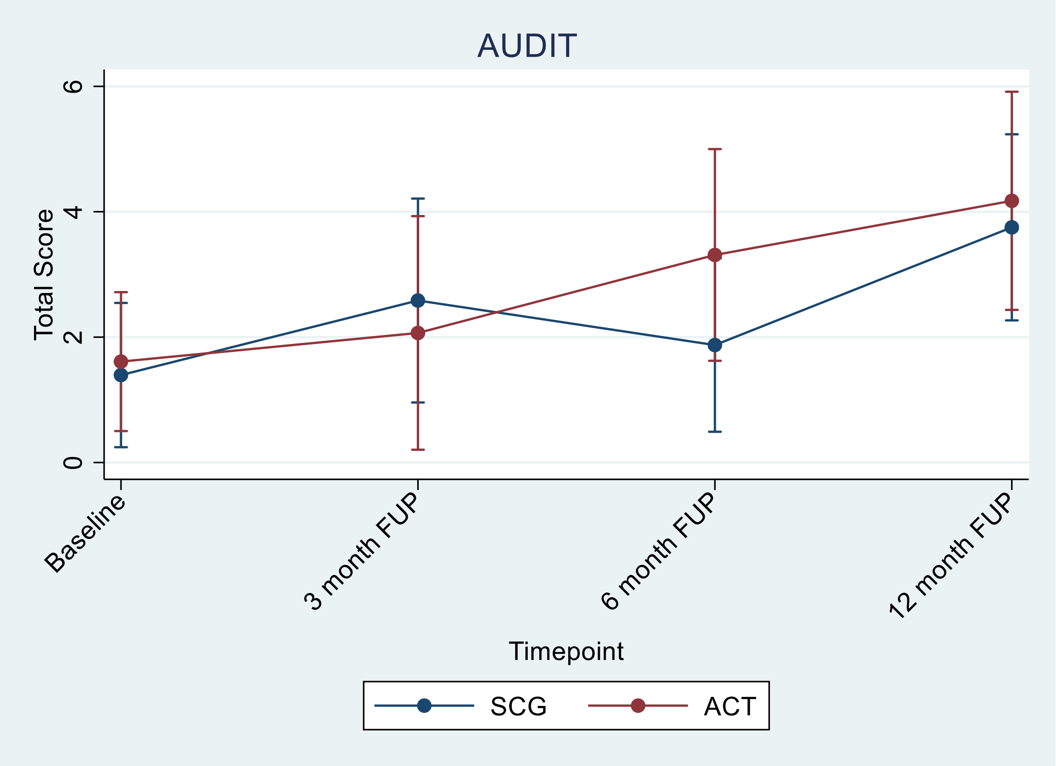

A high score is indicative of unhealthy alcohol consumption

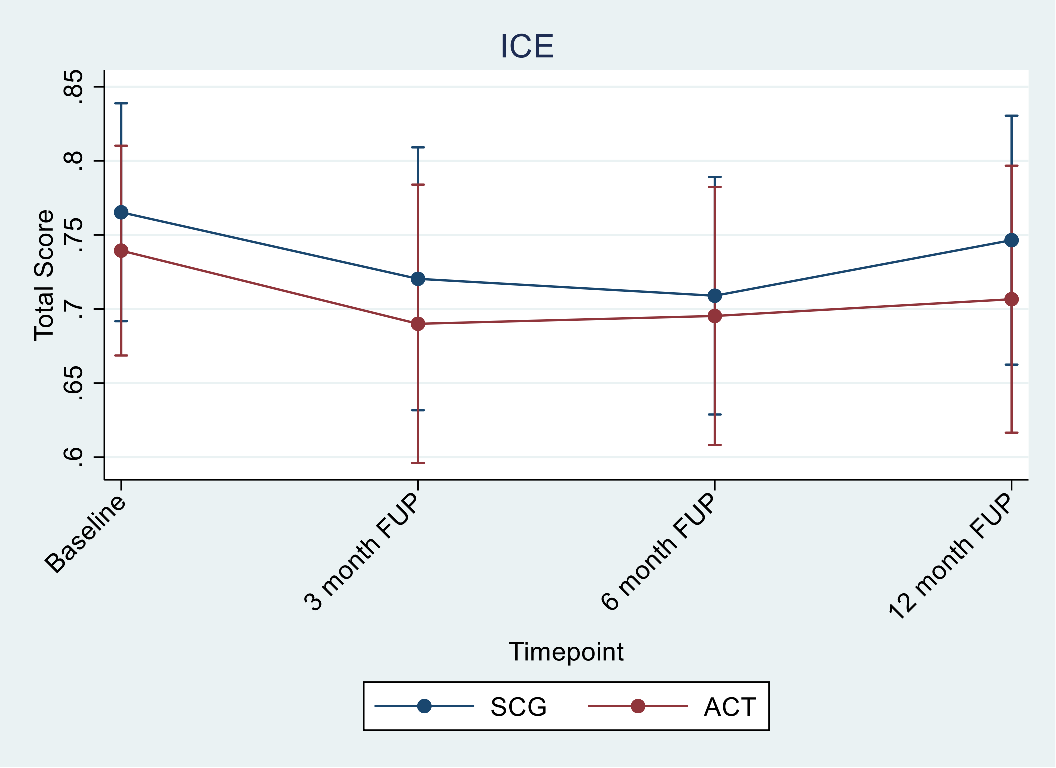

A higher score indicates a better quality of life

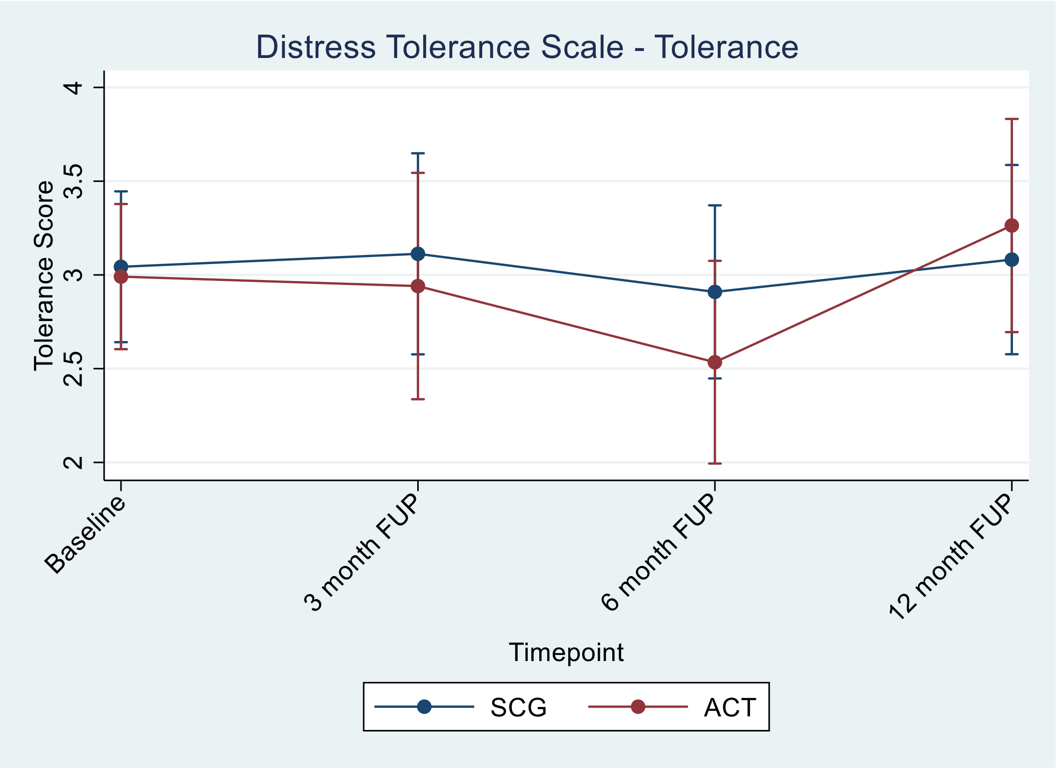

A higher score indicates a greater tolerance to distress

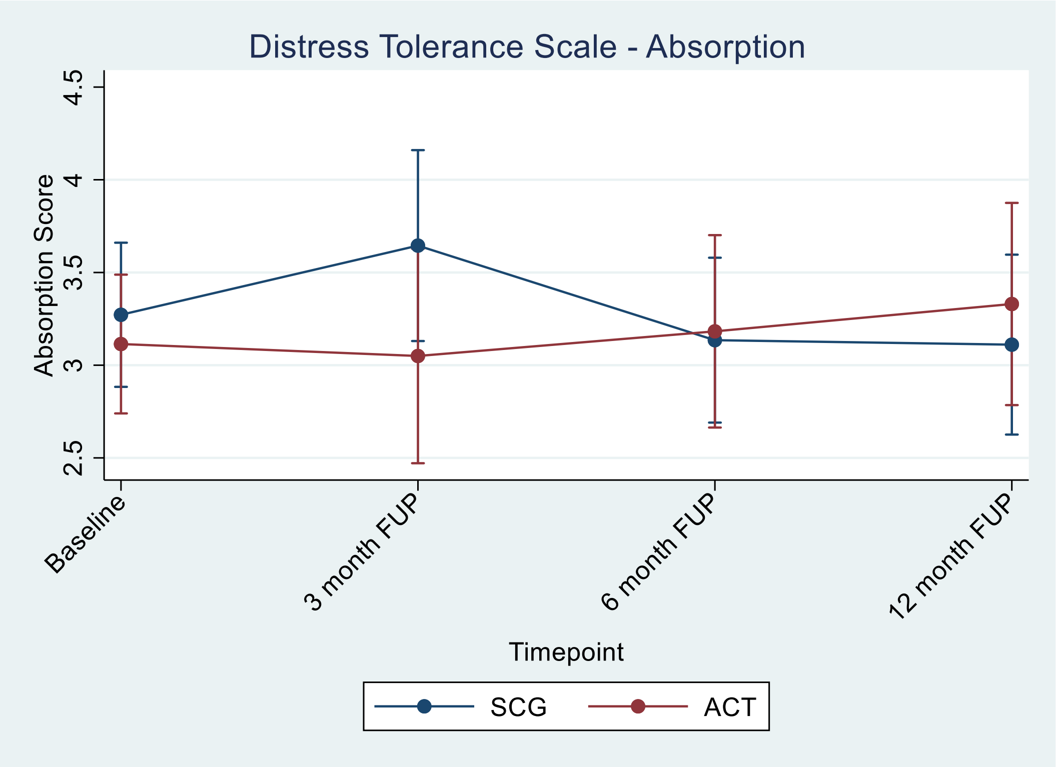

A higher score indicates less absorption in distressing feelings

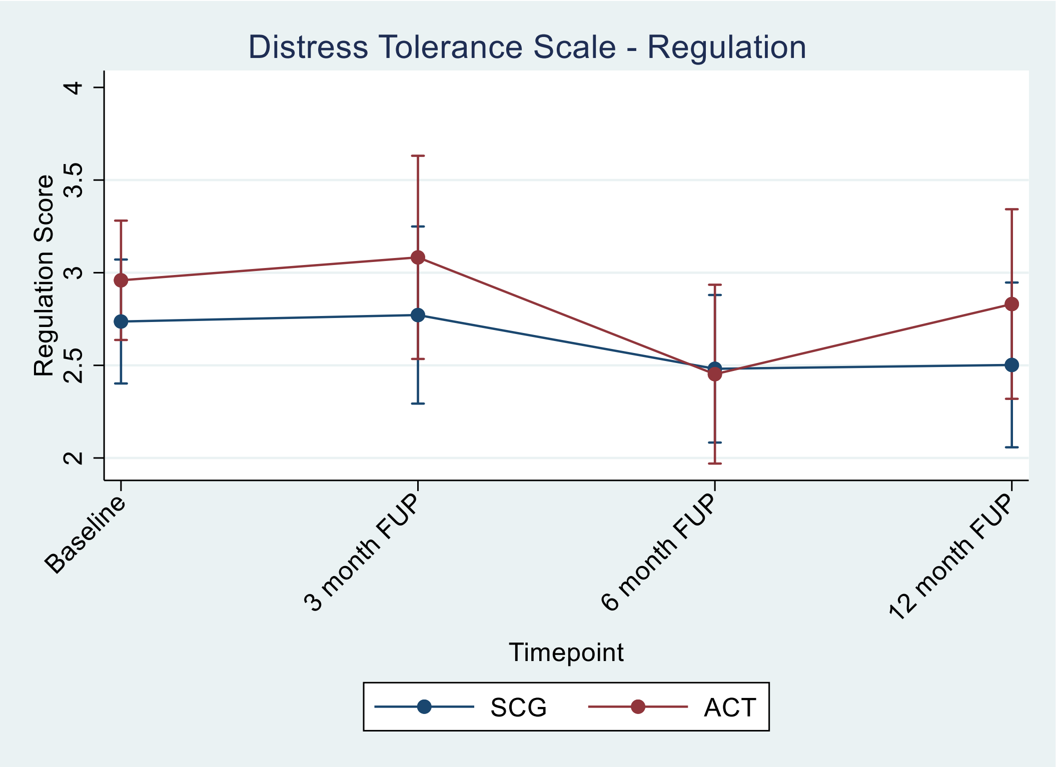

A higher score indicates better regulation of distress

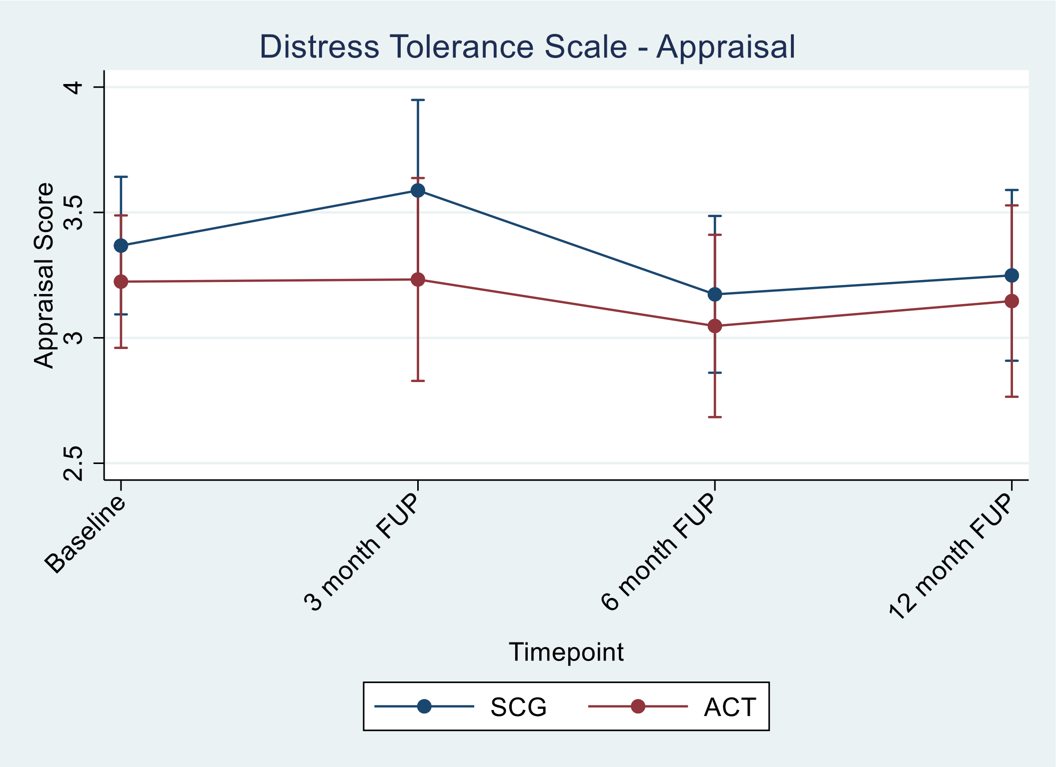

A higher score indicates a greater ability to appraise

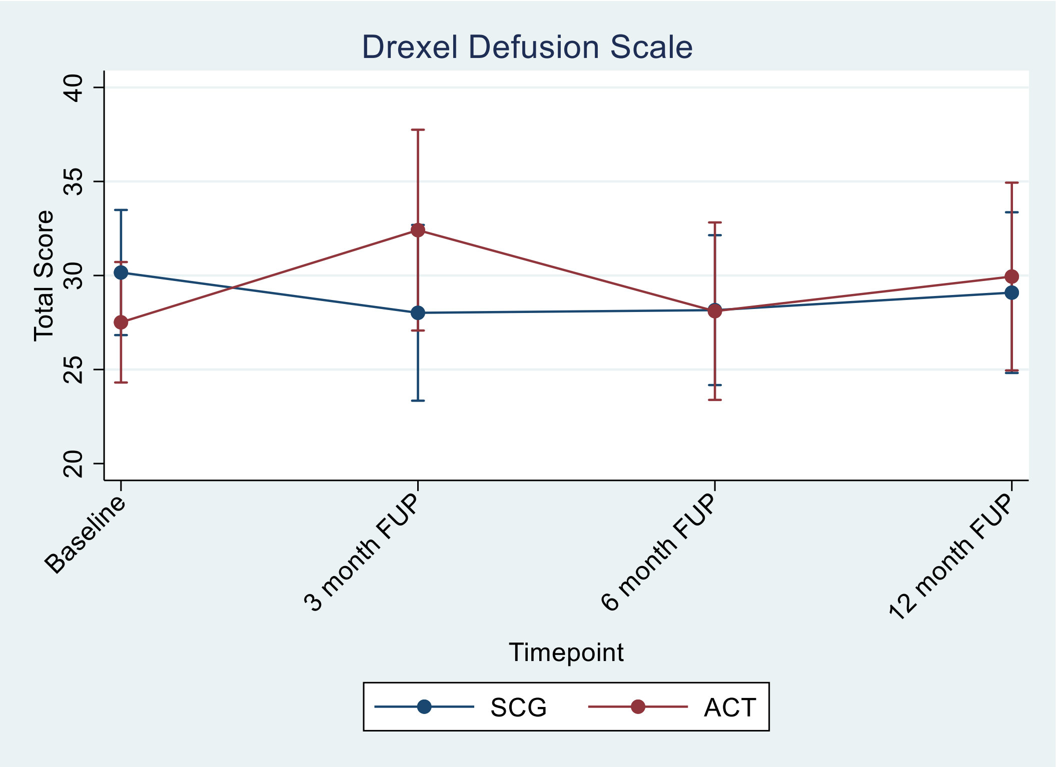

A higher score indicates a greater ability to defuse feelings

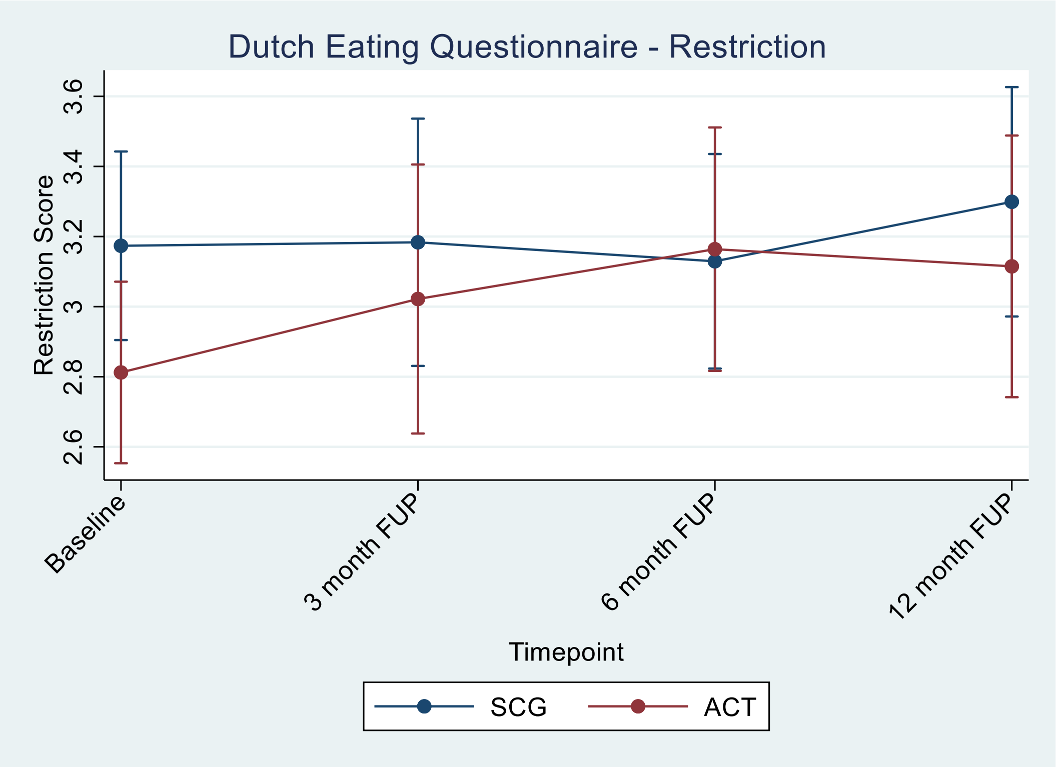

A higher score indicates more food restriction

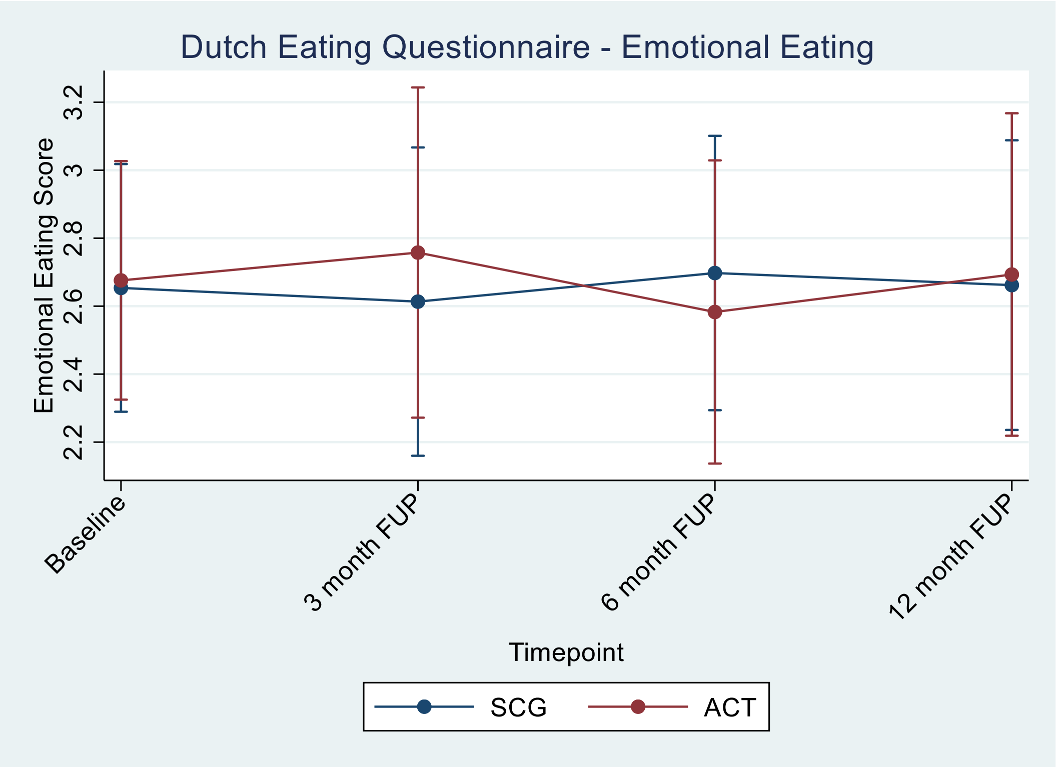

A higher score indicates more emotional eating triggers

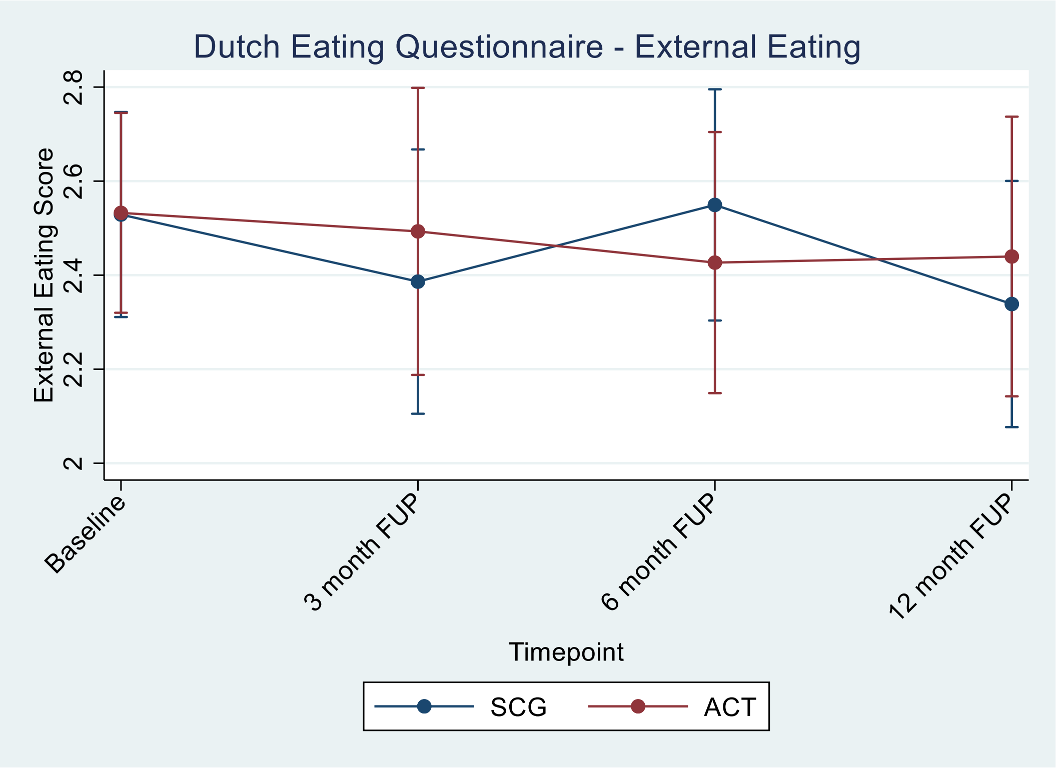

A higher score indicates more external eating triggers

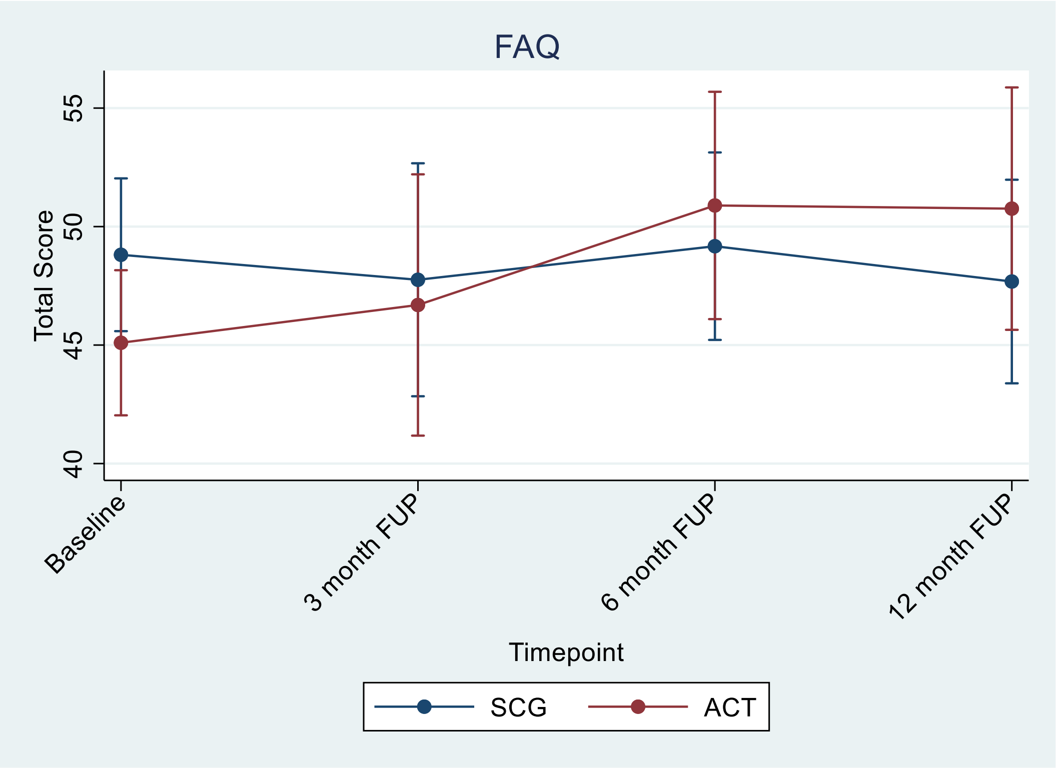

A higher score indicates greater acceptance

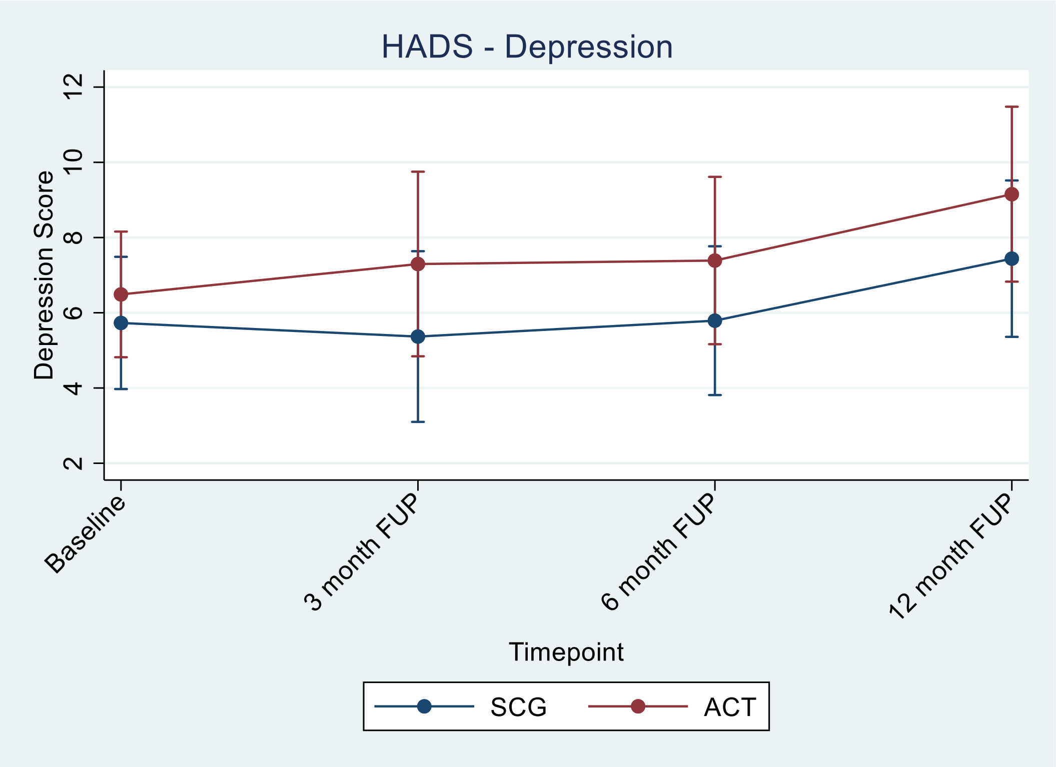

Higher scores indicate depression

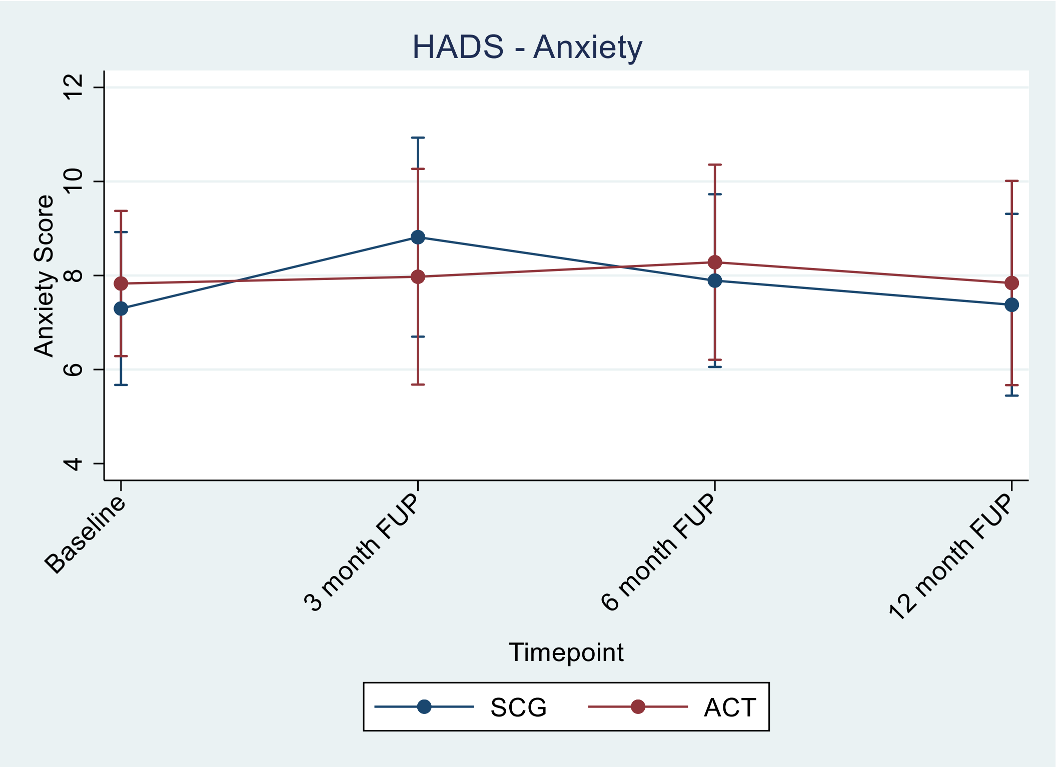

Higher scores indicate anxiety

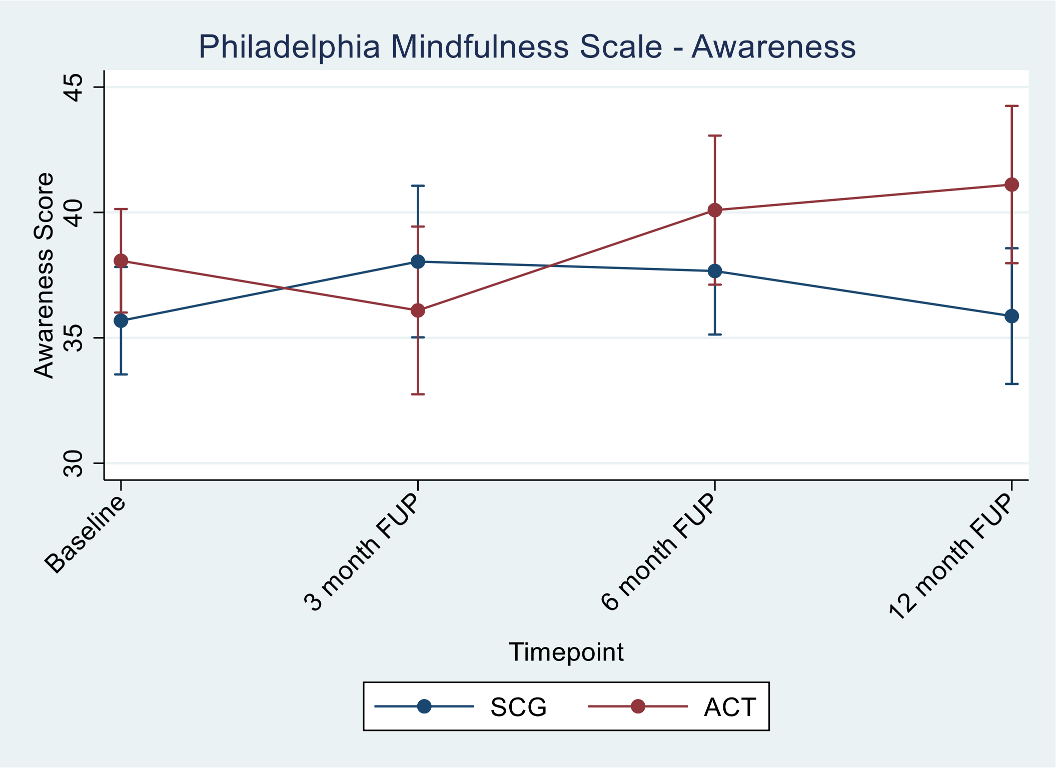

Higher scores indicate greater awareness; p-value (interaction) 0.04

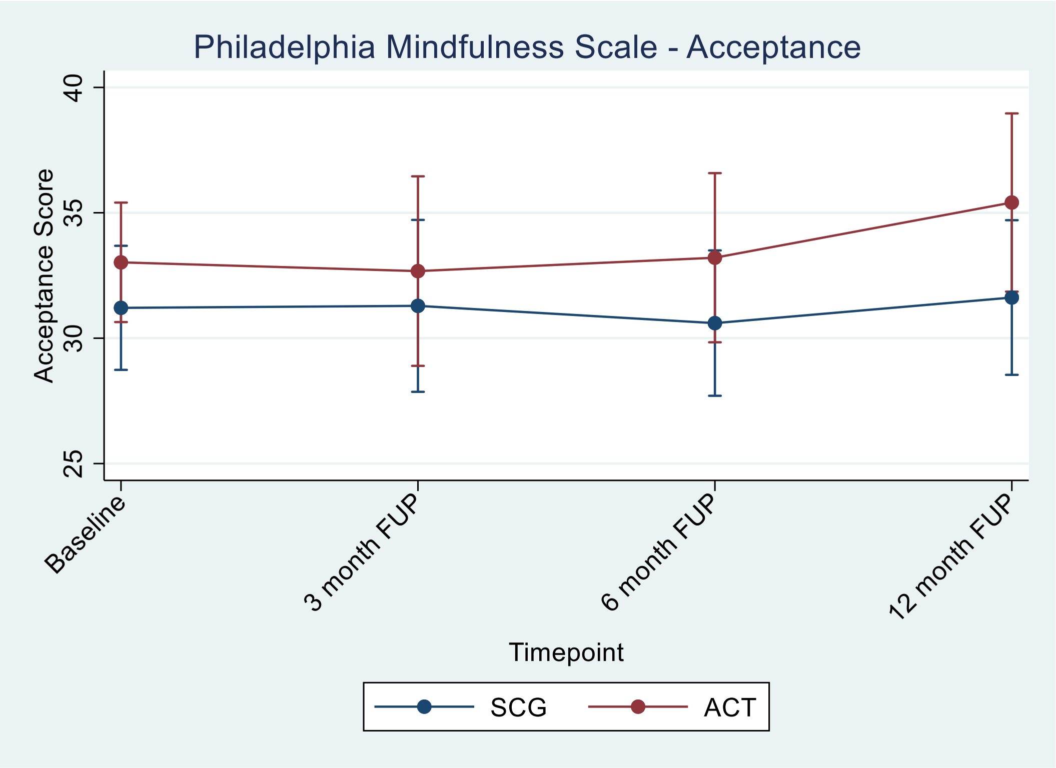

Higher scores indicate less acceptance

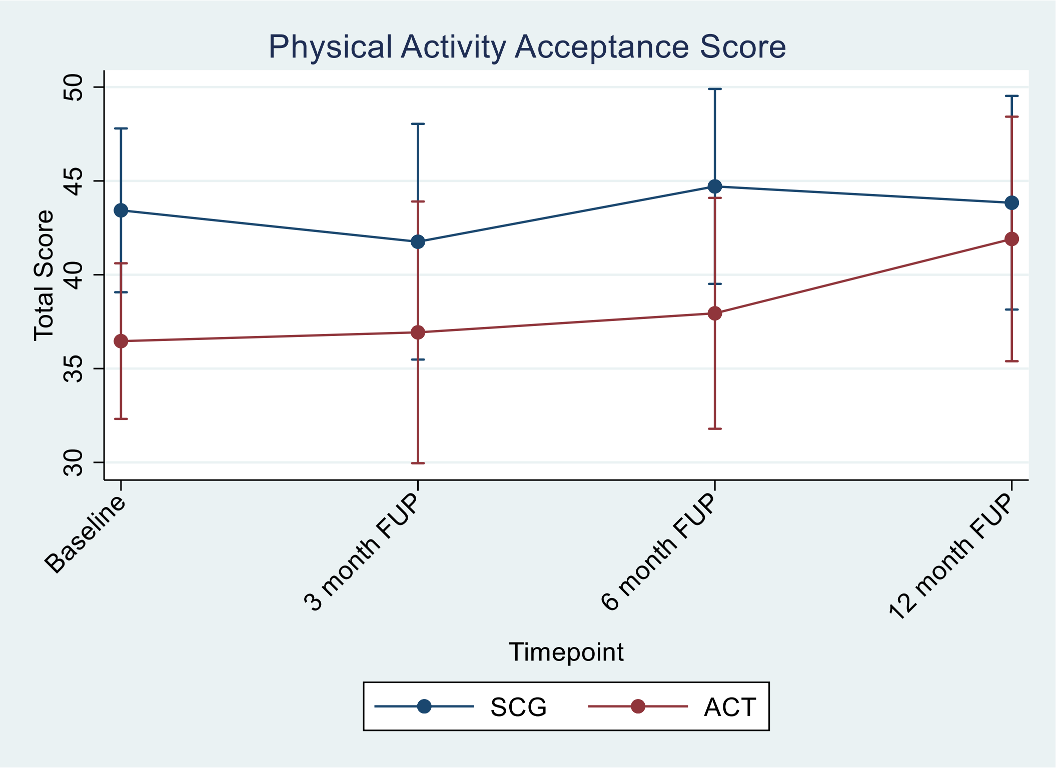

Higher scores indicate greater acceptance; p-value (main effect) 0.02
