# Supplementary material for: Addressing a critical need: A randomised controlled feasibility trial of acceptance and commitment therapy for bariatric surgery patients at 15–18 months post-surgery
Source: PLoS One. 2023 Apr 25;18(4):e0282849. doi: 10.1371/journal.pone.0282849 (PMC10128967; doi:10.1371/journal.pone.0282849)
Supplement: S1 Table — (PDF) [file pone.0282849.s005.pdf]

S2 Table. Questionnaire data

Questionnaire Response Rates

	**SCG**	**ACT**	**Total**	**P-value**
Number randomised n (%)	38	42	80	
Q3month, n (%)				
Still in study	**37**	**34**	**71**	
Completed	12 (32.4)	9 (26.5)	21 (29.6)	0.76
Not completed	25 (67.6)	25 (73.5)	50 (70.4)	
Q6 month, n (%)				
Still in study	**37**	**32**	**69**	
Completed	20 (54.1)	14 (43.8)	34 (49.3)	0.55
Not completed	17 (45.9)	18 (56.2)	35 (50.7)	
Q12month, n (%)				
Still in study	**37**	**31**	**68**	
Completed	19 (51.4)	14 (45.2)	33 (48.5)	0.70
Not completed	19 (48.6)	17 (54.8)	36 (51.5)	
Completed at 6-month and 12-month follow-ups	15 (40.5)	8 (25.8)	23 (33.8)	

There was a problem collecting the data at 3 months, hence the low rates in both groups. However, this was rectified by the 6 month follow up period, where we see higher rates of return for SCG compared with the ACT group, although this was not statistically significant (p=0.55).
