# Supplementary material for: Addressing a critical need: A randomised controlled feasibility trial of acceptance and commitment therapy for bariatric surgery patients at 15–18 months post-surgery
Source: PLoS One. 2023 Apr 25;18(4):e0282849. doi: 10.1371/journal.pone.0282849 (PMC10128967; doi:10.1371/journal.pone.0282849)
Supplement: S2 Table — (PDF) [file pone.0282849.s006.pdf]

S3 Table. Questionnaire results

**Baseline**		**SCG**	**ACT**	**Total**
n (%)		38 (47.5)	42 (52.5)	80 (100.0)
**AUDIT total score**	median (min; max)	1.0 (0.0; 7.0)	(0.0; 10.0)	(0.0; 10.0)
	Missing, n (%)	0	1 (2.4)	
**ICECAP questionnaire**				
Feeling settled and secure, n (%)	Level 1	4 (10.5)	6 (14.6)	10 (12.7)
	Level 2	9 (23.7)	10 (24.4)	19 (24.1)
	Level 3	15 (39.5)	18 (43.9)	33 (41.8)
	Level 4	10 (26.3)	7 (17.1)	17 (21.5)
	Missing, n (%)	0	1 (2.4)	
Love, friendship, and support, n (%)	Level 1	1 (2.6)	0 (0.0)	1 (1.3)
	Level 2	12 (31.6)	10 (24.4)	22 (27.8)
	Level 3	9 (23.7)	11 (26.8)	20 (25.3)
	Level 4	16 (42.1)	20 (48.8)	36 (45.6)
	Missing, n (%)	0	1 (2.4)	
Being independent, n (%)	Level 1	1 (2.6)	2 (4.9)	3 (3.8)
	Level 2	8 (21.1)	9 (22.0)	17 (21.5)
	Level 3	9 (23.7)	12 (29.3)	21 (26.6)
	Level 4	20 (52.6)	18 (43.9)	38 (48.1)
	Missing, n (%)	0	1 (2.4)	
Achievement and progress, n (%)	Level 1	2 (5.3)	3 (7.3)	5 (6.3)
	Level 2	7 (18.4)	11 (26.8)	18 (22.8)
	Level 3	18 (47.4)	19 (46.3)	37 (46.8)
	Level 4	11 (28.9)	8 (19.5)	19 (24.1)
	Missing, n (%)	0	1 (2.4)	
Enjoyment and pleasure,	Level 1	0 (0.0)	2 (4.9)	2 (2.5)

Baseline		SCG	ACT	Total
n (%)	Level 2	11 (28.9)	13 (31.7)	24 (30.4)
	Level 3	15 (39.5)	16 (39.0)	31 (39.2)
	Level 4	12 (31.6)	10 (24.4)	22 (27.8)
	Missing, n (%)	0	1 (2.4)	
ICE total score		0.8 (0.2)	0.7 (0.2)	0.8 (0.2)
	Missing, n (%)	0	1 (2.4)	
**Mediterranean Diet Questionnaire total score**	mean (sd)	7.4 (1.9)	7.1 (2.4)	7.3 (2.2)
	Missing, n (%)	0	1 (2.4)	
**Distress Tolerance Scale**				
Tolerance	mean (sd)	3.0 (1.4)	3.0 (1.2)	3.0 (1.3)
	Missing, n (%)	0	1 (2.4)	
Absorption	mean (sd)	3.3 (1.2)	3.1 (1.3)	3.2 (1.2)
	Missing, n (%)	0	1 (2.4)	
Appraisal	mean (sd)	3.4 (1.0)	3.2 (0.8)	3.3 (0.9)
	Missing, n (%)	0	1 (2.4)	
Regulation	mean (sd)	2.7 (1.2)	3.0 (1.1)	2.9 (1.2)
	Missing, n (%)	0	1 (2.4)	
**Drexel Defusion Scale total score**	mean (sd)	30.2 (9.3)	27.5 (11.5)	28.8 (10.5)
	Missing, n (%)	0	1 (2.4)	
**DUTCH eating questionnaire**				
Restriction	mean (sd)	3.2 (0.9)	2.8 (0.8)	3.0 (0.9)
	Missing, n (%)	0	1 (2.4)	
Emotional eating	mean (sd)	2.7 (1.1)	2.7 (1.2)	2.7 (1.2)
	Missing, n (%)	0	1 (2.4)	
External eating	mean (sd)	2.5 (0.7)	2.5 (0.7)	2.5 (0.7)

**Baseline**		**SCG**	**ACT**	**Total**
	Missing, n (%)	0	2 (4.8)	
**FAQ total score**	mean (sd)	48.8 (10.7)	45.1 (10.3)	46.9 (10.6)
	Missing, n (%)	1 (2.6)	1 (2.4)	
**Hospital Anxiety & Depression Scale**				
Depression	mean (sd)	5.7 (5.1)	6.5 (5.4)	6.1 (5.2)
	Missing, n (%)	1	1 (2.4)	
Anxiety	mean (sd)	7.3 (5.6)	7.8 (5.0)	7.6 (5.3)
	Missing	1 (2.6)	1 (2.4)	
**Philadelphia Mindfulness Scale**				
Awareness	mean (sd)	35.7 (7.5)	38.1 (6.1)	36.9 (6.9)
	Missing, n (%)	0	1 (2.4)	
Acceptance	mean (sd)	31.2 (8.6)	33.0 (7.3)	32.2 (8.0)
	Missing, n (%)	0	1 (2.4)	
**PAAQ total**	mean (sd)	43.4 (14.0)	36.5 (14.6)	39.8 (14.7)
	Missing, n (%)	1 (2.6)	1 (2.4)	
**Kings Obesity Questionnaire**<br>Missing data still to be completed				
Airways, n (%)	Normal	27 (79.4)	31 (75.6)	58 (77.3)
	At risk	2 (5.9)	3 (7.3)	5 (6.7)
	Established	5 (14.7)	7 (17.1)	12 (16.0)
BMI, n (%)	Normal	22 (62.9)	19 (46.3)	41 (53.9)
	At risk	4 (11.4)	16 (39.0)	20 (26.3)

Baseline		SCG	ACT	Total
	Established	8 (22.9)	5 (12.2)	13 (17.1)
	Advanced	1 (2.9)	1 (2.4)	2 (2.6)
Cardiovascular, n (%)	Normal	21 (61.8)	22 (53.7)	43 (57.3)
	At risk	10 (29.4)	15 (36.6)	25 (33.3)
	Established	3 (8.8)	3 (7.3)	6 (8.0)
	Advanced	0 (0.0)	1 (2.4)	1 (1.3)
Diabetes, n (%)	Normal	27 (79.4)	26 (63.4)	53 (70.7)
	At risk	5 (14.7)	3 (7.3)	8 (10.7)
	Established	2 (5.9)	12 (29.3)	14 (18.7)
Economic, n (%)	Normal	32 (94.1)	34 (82.9)	66 (88.0)
	At risk	1 (2.9)	1 (2.4)	2 (2.7)
	Established	0 (0.0)	1 (2.4)	1 (1.3)
	Advanced	1 (2.9)	5 (12.2)	6 (8.0)
Functional, n (%)	Normal	29 (85.3)	34 (82.9)	63 (84.0)
	At risk	5 (14.7)	5 (12.2)	10 (13.3)
	Established	0 (0.0)	2 (4.9)	2 (2.7)
Gonadal, n (%)	Normal	31 (91.2)	38 (92.7)	69 (92.0)
	At risk	2 (5.9)	1 (2.4)	3 (4.0)
	Established	0 (0.0)	1 (2.4)	1 (1.3)
Health perceived, n (%)	Normal	25 (73.5)	30 (73.2)	55 (73.3)
	At risk	4 (11.8)	5 (12.2)	9 (12.0)
	Established	3 (8.8)	5 (12.2)	8 (10.7)
	Advanced	2 (5.9)	1 (2.4)	3 (4.0)
Body Image, n (%)	Normal	22 (66.7)	28 (68.3)	50 (67.6)
	At risk	10 (30.3)	8 (19.5)	18 (24.3)
	Established	0 (0.0)	3 (7.3)	3 (4.1)
	Advanced	1 (3.0)	2 (4.9)	3 (4.1)

**3 months**		**SCG**	**ACT**	**Total**	**p-value***
Number randomised		38 (47.5)	42 (52.5)	80 (100.0)	
Still in study; n(%) Percentage of randomised		37 (97)	34 (81)	71 (89)	
**AUDIT total score**	Responses; n(%) Percentage of those still in study	15 (40.5)	11 (32.4)	26 (36.6)	
	mean (sd)				
	median (min; max)	(0.0; 23.0)	0.0 (0.0; 9.0)	(0.0; 23.0)	
	Missing, n (%)	*22 (59.5)*	*23 (67.6)*		
**ICECAP questionnaire**	Responses; n(%) Percentage of those still in study	14 (37.8)	11 (32.4)	25 (35.2)	
Feeling settled and secure, n (%)	Level 1	1 (7.1)	3 (27.3)	4 (16.0)	0.32
	Level 2	7 (50.0)	4 (36.4)	11 (44.0)	
	Level 3	4 (28.6)	4 (36.4)	8 (32.0)	
	Level 4	2 (14.3)	0 (0.0)	2 (8.0)	
	Missing, n (%)	*23 (62.2)*	*23 (67.6)*		
Love, friendship, and support, n (%)	Level 1	1 (7.1)	1 (9.1)	2 (8.0)	0.57
	Level 2	2 (14.3)	4 (36.4)	6 (24.0)	
	Level 3	3 (21.4)	1 (9.1)	4 (16.0)	
	Level 4	8 (57.1)	5 (45.5)	13 (52.0)	
	Missing, n (%)	*23 (62.2)*	*23 (67.6)*		
Being independent, n (%)	Level 1	0 (0.0)	3 (27.3)	3 (12.0)	0.09
	Level 2	3 (21.4)	4 (36.4)	7 (28.0)	
	Level 3	5 (35.7)	1 (9.1)	6 (24.0)	
	Level 4	6 (42.9)	3 (27.3)	9 (36.0)	
	Missing, n (%)	*23 (62.2)*	*23 (67.6)*		

3 months		SCG	ACT	Total	p-value*
Achievement and progress, n (%)	Level 1	0 (0.0)	3 (27.3)	3 (12.0)	0.05
	Level 2	5 (35.7)	6 (54.5)	11 (44.0)	
	Level 3	7 (50.0)	2 (18.2)	9 (36.0)	
	Level 4	2 (14.3)	0 (0.0)	2 (8.0)	
	Missing, n (%)	*23 (62.2)*	*23 (67.6)*		
Enjoyment and pleasure, n (%)	Level 1	0 (0.0)	2 (18.2)	2 (8.0)	0.40
	Level 2	5 (35.7)	4 (36.4)	9 (36.0)	
	Level 3	4 (28.6)	2 (18.2)	6 (24.0)	
	Level 4	5 (35.7)	3 (27.3)	8 (32.0)	
	Missing, n (%)	*23 (62.2)*	*23 (67.6)*		
ICE total score	mean (sd)	0.8 (0.2)	0.7 (0.2)	0.8 (0.2)	
	Missing, n (%)	*23 (62.2)*	*23 (67.6)*		
**Mediterranean Diet Questionnaire total score**	Responses; n(%) Percentage of those still in study	15 (40.5)	11 (32.4)	26 (36.6)	
	mean (sd)	7.5 (1.6)	8.6 (1.8)	8.0 (1.8)	0.12
	Missing, n (%)	*22 (59.5)*	*23 (67.6)*		
**Distress Tolerance Scale**	Responses; n(%) Percentage of those still in study	15 (40.5)	11 (32.4)	26 (36.6)	
Tolerance	mean (sd)	3.1 (1.4)	3.1 (1.2)	3.1 (1.3)	0.89
	Missing, n (%)	*22 (59.5)*	*23 (67.6)*		
Absorption	mean (sd)	3.0 (1.4)	2.9 (1.1)	3.0 (1.3)	0.68
	Missing, n (%)	*22 (59.5)*	*23 (67.6)*		
Appraisal	mean (sd)	3.8 (0.6)	3.0 (0.8)	3.5 (0.8)	0.02
	Missing, n (%)	*22 (59.5)*	*23 (67.6)*		
Regulation	mean (sd)	2.9 (1.1)	3.1 (0.9)	3.0 (1.0)	0.62
	Missing, n (%)	*22 (59.5)*	*23 (67.6)*		

3 months		SCG	ACT	Total	p-value*
**Drexel Defusion Scale total score**	Responses; n(%)<br>Percentage of those still in study	15 (40.5)	11 (32.4)	26 (36.6)	
	mean (sd)	29.0 (10.3)	30.8 (9.2)	29.8 (9.7)	0.65
	Missing, n (%)	*22 (59.5)*	*23 (67.6)*		
**DUTCH eating questionnaire**	Responses; n(%)<br>Percentage of those still in study	14 (37.8)	11 (32.4)	25 (35.2)	
Restriction	mean (sd)	3.1 (1.1)	2.9 (0.5)	3.0 (0.9)	0.57
	Missing, n (%)	*23 (62.2)*	*23 (67.6)*		
Emotional eating	mean (sd)	2.4 (1.1)	2.8 (1.3)	2.6 (1.2)	0.42
	Missing, n (%)	*23 (62.2)*	*23 (67.6)*		
External eating	mean (sd)	2.4 (0.8)	2.5 (0.8)	2.4 (0.8)	0.61
	Missing, n (%)	*23 (62.2)*	*23 (67.6)*		
**FAQ total score**	Responses; n(%)<br>Percentage of those still in study	14 (37.8)	11 (32.4)	25 (35.2)	
	mean (sd)	48.1 (10.1)	45.5 (12.0)	47.0 (10.8)	0.56
	Missing, n (%)	*23 (62.2)*	*23 (67.6)*		
**Hospital Anxiety & Depression Scale**	Responses; n(%)<br>Percentage of those still in study	14 (37.8)	11 (32.4)	25 (35.2)	
Depression	mean (sd)	4.4 (3.5)	8.5 (6.4)	6.2 (5.3)	0.05
	Missing, n (%)	*23 (62.2)*	*23 (67.6)*		
Anxiety	mean (sd)	7.9 (3.7)	9.7 (5.3)	8.7 (4.5)	0.33
	Missing, n (%)	*23 (62.2)*	*23 (67.6)*		

3 months		SCG	ACT	Total	p-value*
**Philadelphia Mindfulness Scale**	Responses; n(%) Percentage of those still in study	14 (37.8)	11 (32.4)	25 (35.2)	
Awareness	mean (sd)	37.1 (9.2)	33.7 (5.8)	35.6 (7.9)	0.29
	Missing, n (%)	*23 (62.2)*	*23 (67.6)*		
Acceptance	mean (sd)	30.0 (8.2)	32.4 (7.0)	31.0 (7.6)	0.45
	Missing, n (%)	*23 (62.2)*	*23 (67.6)*		
**PAAQ total**	Responses; n(%) Percentage of those still in study	14 (37.8)	11 (32.4)	25 (35.2)	
	mean (sd)	41.1 (13.8)	34.5 (11.6)	38.2 (13.1)	0.21
	Missing, n (%)	*23 (62.2)*	*23 (67.6)*		

*p-values are for differences between groups, from a t-test where means are reported, Wilcoxon rank-sum test for medians, and chi-squared for categorical variables.

6 months		SCG	ACT	Total	p-value*
Number randomised		38 (47.5)	42 (52.5)	80 (100.0)	
Still in study; n(%) Percentage of randomised		37 (97)	32 (76)	69 (86)	
**AUDIT total score**	Responses; n(%) Percentage of those still in study	23 (62.2)	14 (43.8)	37 (53.6)	
	median (min; max)	1.0 (0.0; 6.0)	(0.0; 24.0)	(0.0; 24.0)	0.81
	Missing, n (%)	*14 (37.8)*	*18 (56.2)*		
**ICECAP questionnaire**					
	Responses; n(%) Percentage of those still in study	23 (62.2)	15 (53.1)	38 (55.1)	

6 months		SCG	ACT	Total	p-value*
Feeling settled and secure, n (%)	Level 1	4 (17.4)	0 (0.0)	4 (10.5)	0.03
	Level 2	11 (47.8)	8 (53.3)	19 (50.0)	
	Level 3	2 (8.7)	6 (40.0)	8 (21.1)	
	Level 4	6 (26.1)	1 (6.7)	7 (18.4)	
	Missing, n (%)	*14 (37.8)*	*17 (53.1)*		
Love, friendship, and support, n (%)	Level 1	2 (8.7)	1 (6.7)	3 (7.9)	0.99
	Level 2	8 (34.8)	5 (33.3)	13 (34.2)	
	Level 3	8 (34.8)	5 (33.3)	13 (34.2)	
	Level 4	5 (21.7)	4 (26.7)	9 (23.7)	
	Missing, n (%)	*14 (37.8)*	*17 (53.1)*		
Being independent, n (%)	Level 1	1 (4.3)	1 (6.7)	2 (5.3)	0.57
	Level 2	7 (30.4)	4 (26.7)	11 (28.9)	
	Level 3	8 (34.8)	8 (53.3)	16 (42.1)	
	Level 4	7 (30.4)	2 (13.3)	9 (23.7)	
	Missing, n (%)	*14 (37.8)*	*17 (53.1)*		
Achievement and progress, n (%)	Level 1	0 (0.0)	1 (6.7)	1 (2.6)	0.65
	Level 2	11 (47.8)	7 (46.7)	18 (47.4)	
	Level 3	8 (34.8)	5 (33.3)	13 (34.2)	
	Level 4	4 (17.4)	2 (13.3)	6 (15.8)	
	Missing, n (%)	*14 (37.8)*	*17 (53.1)*		
Enjoyment and pleasure, n (%)	Level 1	3 (13.0)	1 (6.7)	4 (10.5)	0.90
	Level 2	7 (30.4)	5 (33.3)	12 (31.6)	
	Level 3	9 (39.1)	7 (46.7)	16 (42.1)	
	Level 4	4 (17.4)	2 (13.3)	6 (15.8)	
	Missing, n (%)	*14 (37.8)*	*17 (53.1)*		
ICE total score	mean (sd)	0.6 (0.3)	0.7 (0.2)	0.7 (0.2)	
	Missing, n (%)	*14 (37.8)*	*17 (53.1)*		
	Responses; n(%)	23 (62.2)	14 (43.8)	37 (53.6)	

6 months		SCG	ACT	Total	p-value*
**Mediterranean Diet Questionnaire total score**	Percentage of those still in study				
	mean (sd)	7.8 (2.5)	9.1 (1.6)	8.3 (2.2)	0.10
	Missing, n (%)	*14 (37.8)*	*17 (53.1)*		
**Distress Tolerance Scale**	Responses; n(%) Percentage of those still in study	24 (64.9)	15 (46.9)	39 (76.8)	
Tolerance	mean (sd)	2.8 (1.2)	2.4 (1.0)	2.6 (1.1)	0.21
	Missing, n (%)	*14 (37)*	*27 (64)*		
Absorption	mean (sd)	3.0 (1.4)	2.9 (1.1)	3.0 (1.3)	0.68
	Missing, n (%)	*14 (37)*	*27 (64)*		
Appraisal	mean (sd)	3.0 (1.0)	2.9 (0.9)	3.0 (1.0)	0.74
	Missing, n (%)	*14 (37)*	*27 (64)*		
Regulation	mean (sd)	2.4 (0.9)	2.4 (1.0)	2.4 (0.9)	0.99
	Missing, n (%)	*14 (37)*	*27 (64)*		
**Drexel Defusion Scale total score**	Responses; n(%) Percentage of those still in study	23 (62.2)	15 (53.1)	38 (55.1)	
	mean (sd)	27.3 (11.3)	28.3 (11.5)	27.7 (11.3)	0.80
	Missing, n (%)	*14 (37.8)*	*17 (53.1)*		
**DUTCH eating questionnaire**	Responses; n(%) Percentage of those still in study	23 (62.2)	15 (53.1)	38 (55.1)	
Restriction	mean (sd)	3.1 (0.9)	3.3 (0.8)	3.2 (0.9)	0.42
	Missing, n (%)	*14 (37.8)*	*17 (53.1)*		
Emotional eating	mean (sd)	2.7 (1.1)	2.7 (1.2)	2.7 (1.1)	0.97
	Missing, n (%)	*14 (37.8)*	*17 (53.1)*		

6 months		SCG	ACT	Total	p-value*
External eating	mean (sd)	2.5 (0.6)	2.4 (0.7)	2.5 (0.6)	0.70
	Missing, n (%)	*14 (37.8)*	*17 (53.1)*		
**FAQ total score**	Responses; n(%) Percentage of those still in study	23 (62.2)	15 (53.1)	38 (55.1)	
	mean (sd)	49.0 (9.7)	51.1 (8.4)	49.9 (9.2)	0.50
	Missing, n (%)	*14 (37.8)*	*17 (53.1)*		
**Hospital Anxiety & Depression Scale**	Responses; n(%) Percentage of those still in study	23 (62.2)	15 (53.1)	38 (55.1)	
Depression	mean (sd)	6.5 (6.2)	7.7 (5.9)	7.0 (6.0)	0.58
	Missing, n (%)	*14 (37.8)*	*17 (53.1)*		
Anxiety	mean (sd)	8.6 (4.9)	8.9 (4.6)	8.7 (4.7)	0.85
	Missing, n (%)	*14 (37.8)*	*17 (53.1)*		
**Philadelphia Mindfulness Scale**	Responses; n(%) Percentage of those still in study	23 (62.2)	15 (53.1)	38 (55.1)	
Awareness	mean (sd)	37.6 (7.2)	39.3 (6.4)	38.2 (6.9)	0.46
	Missing, n (%)	14 (37.8)	17 (53.1)		
Acceptance	mean (sd)	31.1 (7.6)	33.3 (8.0)	32.0 (7.7)	0.41
	Missing, n (%)	14 (37.8)	17 (53.1)		
	Responses; n(%) Percentage of those still in study	23 (62.2)	15 (53.1)	38 (55.1)	
**PAAQ total**	mean (sd)	44.6 (11.0)	37.5 (13.0)	41.8 (12.2)	0.08
	Missing, n (%)	*14 (37.8)*	*17 (53.1)*		

*p-values are for differences between groups, from a t-test where means are reported, Wilcoxon rank-sum test for medians, and chi-squared for categorical variables.

**12 months**		**SCG**	**ACT**	**Total**	**p-value***
Number randomised		38 (47.5)	42 (52.5)	80 (100.0)	
Still in study; n(%)<br>Percentage of those randomised		37 (97)	31 (74)	68 (85)	
**AUDIT total score**	Responses; n(%)<br>Percentage of those still in study	19 (51.4)	13 (41.9)	32 (47.1)	
	median (min; max)	(0.0; 14.0)	(0.0; 20.0)	(0.0; 20.0)	0.66
	Missing, n (%)	*18 (48.6)*	*17 (54.8)*		
**ICECAP questionnaire (% of non-missing)**					
	Responses; n(%)<br>Percentage of those still in study	18 (48.6)	13 (41.9)	31 (45.6)	
Feeling settled and secure,<br>n (%)	Level 1	1 (5.6)	3 (23.1)	4 (12.9)	0.40
	Level 2	7 (38.9)	3 (23.1)	10 (32.3)	
	Level 3	7 (38.9)	6 (46.2)	13 (41.9)	
	Level 4	3 (16.7)	1 (7.7)	4 (12.9)	
	Missing, n (%)	*20 (53)*	*29 (69)*		
Love, friendship, and support,<br>n (%)	Level 1	0 (0.0)	2 (15.4)	2 (6.5)	
	Level 2	5 (27.8)	2 (15.4)	7 (22.6)	
	Level 3	4 (22.2)	3 (23.1)	7 (22.6)	
	Level 4	9 (50.0)	6 (46.2)	15 (48.4)	0.35
	Missing, n (%)	*20 (53)*	*29 (69)*		
Being independent,<br>n (%)	Level 1	0 (0.0)	1 (7.7)	1 (3.2)	
	Level 2	3 (16.7)	1 (7.7)	4 (12.9)	
	Level 3	7 (38.9)	6 (46.2)	13 (41.9)	
	Level 4	8 (44.4)	5 (38.5)	13 (41.9)	0.57

12 months		SCG	ACT	Total	p-value*
	Missing, n (%)	*20 (53)*	*29 (69)*		
Achievement and progress, n (%)	Level 1	0 (0.0)	1 (7.7)	1 (3.2)	0.03
	Level 2	7 (38.9)	2 (15.4)	9 (29.0)	
	Level 3	6 (33.3)	10 (76.9)	16 (51.6)	
	Level 4	5 (27.8)	0 (0.0)	5 (16.1)	
	Missing, n (%)	*20 (53)*	*29 (69)*		
Enjoyment and pleasure, n (%)	Level 1	1 (5.6)	2 (15.4)	3 (9.7)	
	Level 2	4 (22.2)	1 (7.7)	5 (16.1)	
	Level 3	7 (38.9)	6 (46.2)	13 (41.9)	
	Level 4	6 (33.3)	4 (30.8)	10 (32.3)	0.60
	Missing, n (%)	*20 (53)*	*29 (69)*		
ICE total score	mean (sd)	0.6 (0.3)	0.7 (0.2)	0.7 (0.2)	
	Missing, n (%)	*20 (53)*	*29 (69)*		
**Mediterranean Diet Questionnaire total score**	Responses; n(%) Percentage of those still in study	19 (51.4)	13 (41.9)	32 (47.1)	
	mean (sd)	7.0 (2.2)	7.2 (1.6)	7.1 (2.0)	0.83
	Missing, n (%)	*20 (53)*	*29 (69)*		
**Distress Tolerance Scale**					
	Responses; n(%) Percentage of those still in study	18 (48.6)	13 (41.9)	31 (45.6)	
Tolerance	mean (sd)	3.1 (1.4)	3.1 (1.2)	3.1 (1.3)	0.89
	Missing, n (%)	*20 (53)*			
Absorption	mean (sd)	3.2 (1.5)	3.1 (1.0)	3.1 (1.3)	0.90
	Missing, n (%)	*20 (53)*			
Appraisal	mean (sd)	3.2 (0.8)	3.1 (0.9)	3.2 (0.8)	0.57
	Missing, n (%)	*20 (53)*	*29 (69)*		

12 months		SCG	ACT	Total	p-value*
Regulation	mean (sd)	2.4 (1.0)	2.7 (0.9)	2.5 (0.9)	0.34
	Missing, n (%)	*20 (53)*	*29 (69)*		
**Drexel Defusion Scale total score**	Responses; n(%) Percentage of those still in study	19 (51.4)	13 (41.9)	32 (47.1)	
	mean (sd)	27.8 (12.6)	28.9 (6.9)	28.3 (10.5)	0.77
	Missing, n (%)	*19 (50)*	*29 (69)*		
**DUTCH eating questionnaire**					
	Responses; n(%) Percentage of those still in study	18 (48.6)	12 (38.7)	30 (44.1)	
Restriction	mean (sd)	3.2 (0.9)	3.0 (0.8)	3.1 (0.8)	0.55
	Missing, n (%)	*20 (53)*	*30 (71)*		
Emotional eating	mean (sd)	2.6 (1.2)	2.5 (1.2)	2.6 (1.2)	0.87
	Missing, n (%)	*20 (53)*	*30 (71)*		
External eating	mean (sd)	2.2 (0.4)	2.3 (0.8)	2.3 (0.6)	0.78
	Missing, n (%)	*20 (53)*	*30 (71)*		
**FAQ total score**	Responses; n(%) Percentage of those still in study	19 (51.4)	13 (41.9)	32 (47.1)	
	mean (sd)	48.5 (9.5)	49.6 (11.4)	49.0 (10.2)	0.77
	Missing, n (%)	*19 (50)*	*29 (69)*		
**Hospital Anxiety & Depression Scale**					
	Responses; n(%) Percentage of those still in study	19 (51.4)	13 (41.9)	32 (47.1)	
Depression	mean (sd)	7.9 (7.2)	8.5 (5.2)	8.1 (6.4)	0.81

12 months		SCG	ACT	Total	p-value*
	Missing, n (%)	*19 (50)*	*29 (69)*		
Anxiety	mean (sd)	7.4 (5.7)	7.2 (5.0)	7.3 (5.3)	0.91
	Missing, n (%)	*19 (50)*	*29 (69)*		
**Philadelphia Mindfulness Scale**					
	Responses; n(%)<br>Percentage of those still in study	19 (51.4)	13 (41.9)	32 (47.1)	
Awareness	mean (sd)	36.8 (6.7)	40.6 (6.6)	38.4 (6.8)	0.13
	Missing, n (%)	*19 (50)*	*29 (69)*		
Acceptance	mean (sd)	30.9 (8.3)	35.6 (8.0)	32.8 (8.4)	0.12
	Missing, n (%)	*19 (50)*	*29 (69)*		
	Responses; n(%)<br>Percentage of those still in study	18 (48.6)	13 (41.9)	38 (55.9)	
**PAAQ total**	Responses; n(%)<br>Percentage of those still in study	18 (48.6)	13 (41.9)	31 (45.6)	
	mean (sd)	43.2 (12.4)	41.6 (17.9)	42.5 (14.7)	0.77
	Missing, n (%)	*20 (53)*	*29 (69)*		
**Kings Obesity Questionnaire (% of non-missing)**<br>Missing data still to be completed					
Airways, n (%)	Normal	22 (73.3)	31 (81.6)	53 (77.9)	
	At risk	4 (13.3)	2 (5.3)	6 (8.8)	
	Established	4 (13.3)	5 (13.2)	9 (13.2)	0.50
BMI, n (%)	Normal	17 (56.7)	25 (65.8)	42 (61.8)	
	At risk	5 (16.7)	9 (23.7)	14 (20.6)	
	Established	7 (23.3)	3 (7.9)	10 (14.7)	
	Advanced	1 (3.3)	1 (2.6)	2 (2.9)	0.34
Cardiovascular, n (%)	Normal	21 (70.0)	22 (57.9)	43 (63.2)	

12 months		SCG	ACT	Total	p-value*
	At risk	6 (20.0)	14 (36.8)	20 (29.4)	
	Established	3 (10.0)	1 (2.6)	4 (5.9)	
	Advanced	0 (0.0)	1 (2.6)	1 (1.5)	0.23
Diabetes, n (%)	Normal	27 (90.0)	26 (68.4)	53 (77.9)	
	At risk	1 (3.3)	4 (10.5)	5 (7.4)	
	Established	2 (6.7)	8 (21.1)	10 (14.7)	0.10
Economic, n (%)	Normal	29 (96.7)	30 (78.9)	59 (86.8)	
	At risk	0 (0.0)	1 (2.6)	1 (1.5)	
	Advanced	1 (3.3)	7 (18.4)	8 (11.8)	0.10
Functional, n (%)	Normal	26 (86.7)	28 (73.7)	54 (79.4)	
	At risk	4 (13.3)	6 (15.8)	10 (14.7)	
	Established	0 (0.0)	4 (10.5)	4 (5.9)	0.17
Gonadal, n (%)	Normal	28 (93.3)	32 (84.2)	60 (88.2)	
	At risk	2 (6.7)	3 (7.9)	5 (7.4)	
	Established	0 (0.0)	1 (2.6)	1 (1.5)	
	Advanced	0 (0.0)	2 (5.3)	2 (2.9)	0.46
Health perceived, n (%)	Normal	23 (76.7)	22 (57.9)	45 (66.2)	
	At risk	1 (3.3)	13 (34.2)	14 (20.6)	
	Established	4 (13.3)	3 (7.9)	7 (10.3)	
	Advanced	2 (6.7)	0 (0.0)	2 (2.9)	0.01
Body Image, n (%)	Normal	21 (70.0)	27 (71.1)	48 (70.6)	
	At risk	8 (26.7)	10 (26.3)	18 (26.5)	
	Established	0 (0.0)	1 (2.6)	1 (1.5)	
	Advanced	1 (3.3)	0 (0.0)	1 (1.5)	0.56

*p-values are for differences between groups, from a t-test where means are reported, Wilcoxon rank-sum test for medians, and chi-squared for categorical variables.
