# Supplementary material for: Addressing a critical need: A randomised controlled feasibility trial of acceptance and commitment therapy for bariatric surgery patients at 15–18 months post-surgery
Source: PLoS One. 2023 Apr 25;18(4):e0282849. doi: 10.1371/journal.pone.0282849 (PMC10128967; doi:10.1371/journal.pone.0282849)
Supplement: S3 Table — (PDF) [file pone.0282849.s007.pdf]

S4 Table. Results from multi-level linear regression models

		Coefficient (main effect)	95% CI (main effect)	p-value (main effect)	p-value (interaction)
AUDIT	Total score	0.22	(-1.38, 1.81)	0.79	0.54
Mediterranean Diet	Total score	-0.33	(-1.23, -0.58)	0.48	0.09
ICECAP	Total score	-0.03	-0.13, 0.08)	0.62	0.97
Distress Tolerance Scale	Tolerance	-0.05	(-0.61, 0.51)	0.85	0.55
	Absorption	-0.16	(-0.70, 0.38)	0.57	0.23
	Regulation	0.22	(-0.24, 0.69)	0.35	0.78
	Appraisal	-0.14	(-0.52, 0.24)	0.46	0.81
Drexel Defusion	Total score	-2.65	(-7.26, 1.97)	0.26	0.24
Dutch Eating	Restriction	-0.36	(-0.73, 0.01)	0.06	0.27
	Emotional	0.02	(-0.48, 0.53)	0.93	0.86
	External	0.004	(-0.30, 0.31)	0.98	0.60
FAQ	Total score	-3.71	(-8.16, 0.73)	0.10	0.20
HADS	Depression	0.76	(-1.67, 3.18)	0.54	0.81
	Anxiety	0.53	(-1.71, 2.77)	0.64	0.80
Philadelphia Mindfulness	Awareness	2.39	(-0.59, 5.36)	0.12	0.04
	Acceptance	1.81	(-1.62, 5.25)	0.30	0.80
Physical Activity Acceptance	Total score	-6.97	(-13.0, 0.95)	0.02	0.69
