# Supplementary material for: Addressing a critical need: A randomised controlled feasibility trial of acceptance and commitment therapy for bariatric surgery patients at 15–18 months post-surgery
Source: PLoS One. 2023 Apr 25;18(4):e0282849. doi: 10.1371/journal.pone.0282849 (PMC10128967; doi:10.1371/journal.pone.0282849)
Supplement: S1 File — (PDF) [file pone.0282849.s008.pdf]

S7 Supplementary Information. Service use and costs

The cost of service delivery for ACT includes one hour of clinical psychologist time spread across ten patients.). AT £70 per hour, this works out to £7 per patient per session (i.e. £70 for ten sessions). The training costs equates to about £2.25 per patient (i.e. £450 per year for 200 patients). The total cost of providing ACT services is therefore £72.25 per patient (i.e. psychologist time @£70plus training costs @£2.25).. Service delivery costs for SGC include one hour of assistant psychologist time i.e.£34) spread across ten patients. This works out to £3.4 per patient or £34 for ten sessions. Only 28 patients complete the CSRI questionnaire over the 12 months study period. Table S2.1 shows self-reported service utilisation for the two groups.

S7.1 Table. Health Service Utilisation in ACT and Usual Care Groups.

	Usual care group (n=15)	ACT group (n=13)	p-value
Hospital admission			
Any hospital admission	13%	0%	0.172
Number of nights	0.13	0	0.185
A & E			
Any A & E	20%	7.60%	0.353
Number of A & E visit	0.46	0.07	0.312
Outpatient visit			
Any outpatient visit	26%	46%	0.283
Number of outpatient visit	0.73	1.38	0.431

GP visit			
Any GP visit	40%	69%	0.122
Number of GP visit	1.33	3.23	0.153
General practice nurse visit			
Any general practice nurse visit	27%	46%	0.283
Number general practice nurse visit	0.8	0.85	0.932
Telephone Consultation			
Any telephone consultation	20%	31%	0.512
Number of telephone consultation	1.06	0.69	0.695
District nurse/health visitor			
Any district nurse/health visitor	6%	0%	0.343
Number district nurse/health visitor	0.13	0	0.362
Community mental health worker			
Any community mental health worker	7%	8%	0.916
Number community mental health worker	0.2	0.08	0.592
Psychiatrist			
Any psychiatrist visit	7%	8%	0.916
Number of psychiatrist visit	0.27	0.31	0.921
Social Worker			

Any social worker visit	7%	0%	0.343
Number of social worker visit	0.13	0	0.361
Counselling			
Any counselling	7%	8%	0.916
Number of counselling visit	0.7	0.38	0.391
Art/drama/music therapy in the community			
Any art/drama/music therapy in the community	7%	0%	0.343
Number art/drama/music therapy	0.7	0	0.362
Day centre			
Any day centre visit	7%	38%	0.041
Number of day centre visit	0.13	0.77	0.093
Chiropractor			
Any chiropractor visit	0%	7%	0.274
Number chiropractor visit	0	0.7	0.291

Notes: The Chi-square test was used to determine whether there was a difference between two groups for a binary outcome variable. The T-test was used to compare differences between two groups for a continuous outcome variable.

S 7.2 Table. Service utilisation and intervention cost

	Usual care group (n=15)	ACT group (n=13)
Health service cost (£)		
Hospital admission	40.6	0
Outpatients visit at a hospital	107.8	203.5
A&E use	78.4	12.9
GP visit	73.3	177.6
General practice nurse visit	10.4	11
Telephone consultation	44.8	29.1
District nurse/health visitor	6.3	0
Community mental health worker	7.4	2.8
Psychiatrist visit	52	60
Social worker visit	3.4	0
Counselling	6.4	36.9
Art/drama/music therapy in the community	5.46	0
Day centre visit	4.4	25.3
Chiropractor	0	3.23
Cost of intervention		
Service delivery	34	70

Training cost	0	2.25
Total cost	475	633

Table S7.3 Summary of regression output

	B	95% CI	P-value
Baseline utility	0.98	(0.86,1.09)	0.0005
ACT group (ref usual care)	-0.07	(-0.14, 0.006)	0.072
Age	0.001	(-0.001, 0.004)	0.377
Female (ref male)	-0.02	(-0.116, 0.07)	-0.659
Baseline BMI	-0.001	(-0.005, 0.003)	0.484
