# Supplementary material for: Addressing a critical need: A randomised controlled feasibility trial of acceptance and commitment therapy for bariatric surgery patients at 15–18 months post-surgery
Source: PLoS One. 2023 Apr 25;18(4):e0282849. doi: 10.1371/journal.pone.0282849 (PMC10128967; doi:10.1371/journal.pone.0282849)
Supplement: S2 File — (PDF) [file pone.0282849.s009.pdf]

S8 Protocol. Feasibility of acceptance and commitment therapy for post-bariatric surgery patients: the FAB study protocol

Protocol

Feasibility of acceptance and commitment therapy for post-bariatric surgery patients: the FAB study protocol

Lisa Cotter[1], Samantha Scholtz[2], Shikta Das[3], John Tayu Lee[4], Dayna Lee-Baggley[5], Elizabeth A. Barley[1]*

[1]School of Health Sciences, Kate Granger Building, Guildford, United Kingdom [2]Research and Development, West London Mental Health Trust, Southall, London, United Kingdom [3]UCL Institute of Cardiovascular Science, MRC Unit for Lifelong Health and Ageing at UCL, London, United Kingdom [4]Imperial College London, Reynolds Building, Charing Cross Hospital, London, United Kingdom [5]Behaviour Change Research Institute, Nova Scotia Health Authority, Halifax, Nova Scotia, Canada

ABSTRACT Background: Bariatric surgery is an effective treatment for obesity. However, around one in five people experience significant weight regain. In the months following surgery, loss of food as a hedonic reward, increased sensitivity to food-related cues, alcohol use and depression may translate into new obesogenic behaviours which can be targeted in therapy. Acceptance and Commitment Therapy (ACT) teaches acceptance of and defusion from thoughts and feelings which influence behaviour, and commitment to act in line with personal values. We will test whether people who have had bariatric surgery over one year ago find 10 weeks of ACT group therapy an acceptable treatment and whether a larger trial to test whether ACT can improve long-term post-operative outcomes would be feasible. **Methods:** This will be a feasibility randomised controlled trial (RCT) with participants randomised to either ACT or a Usual Care Support Group control. Participants will be recruited at 15-18 months post-surgery and compared at baseline, 3, 6 and 12 months. The trial will provide information about recruitment and characteristics of the proposed outcome measures to inform a definitive RCT. **Conclusions:** Trials big enough to determine whether a treatment approach works are costly, so this small study will help determine whether the methods used, such as how people are recruited, allocated to groups, and how data are collected, are likely to work on a bigger scale. This project is the first step in testing whether ACT can help people who have had bariatric surgery. **Trial Registration:** Researchregistry.com, UIN: 3959 (date registered: 10 April 2018); ISRCTN registry ID: ISRCTN52074801.

INTRODUCTION

Worldwide, 39% of adults aged 18 years and over are overweight (BMI>25 kg/m^2), and 13% are obese (BMI> 30 kg/m^2).[1] In the UK alone, obesity prevalence rises to 26% and related healthcare costs are £5 to £7 billion per year.[2] Bariatric surgery is an effective treatment for obesity and its comorbidities in the long term; however, up to 20% of patients have significant weight regain which can result in further bariatric procedures, at a cost of £10,000 per re-operation, as well as the return of co-morbidities, such as diabetes.[3,4] Improvements in psychological health post-surgery may also be short-lived and a particular threat to quality of life is the emergence of addictive behaviours such as alcohol dependency, which can occur in as many as nine percent of patients.[5] This has been explained in terms of 'addiction transfer', that is from food to other substances post-operatively, given that food is commonly used for emotional regulation, and this function of eating is largely lost after bariatric surgery.[6] The increased alcohol intake also contributes to post-surgery weight gain.

A number of guidelines suggest that bariatric surgery should be undertaken in the context of a multidisciplinary team that can provide psychological support, though the type of support is not specified.[7] Limited benefit has been found for behavioural interventions which promote healthy lifestyle changes. For instance, a systematic review of 11 trials found small improvements in weight loss at 12 months post-bariatric surgery; however, the included trials were poor quality, short-term and there were few data on outcomes important to patients, such as quality of life.[8] Understanding of the mechanism of action of interventions is also limited. Proposed intervention targets include: loss of food as hedonic reward, emotional regulation, disordered eating, distress intolerance, increased sensitivity to food-related cues, reduced physical activity, alcohol use, and depression.[9] Mindfulness-based or 'third wave' cognitive behaviour therapy approaches such as Acceptance and Commitment therapy (ACT) target these mechanisms and have previously demonstrated effectiveness for weight loss in the general population.[10,11]

In bariatric surgery populations, a small trial (n=39) has demonstrated improvements in an ACT group relative to treatment as usual in areas such as disordered eating, body dissatisfaction, quality of life and acceptance for weight-related thoughts and feelings.[12] Mean time post-surgery in this cohort was 15.5 (range: 4–38) months; however, the optimal timing for psychological intervention is unknown. An RCT (n=162) of a health psychology intervention, which addressed psychological issues such as dietary control, self-esteem, coping and emotional eating, and which was delivered two weeks pre-operatively prior to discharge from hospital, and 3 months post-operatively, had no impact on weight loss at 12 months.[13] Intervention at a later stage may therefore be more beneficial. This makes intuitive sense if it is considered that many of the mechanisms proposed to contribute to weight gain, for example loss of food as a hedonic reward, increased sensitivity to food-related cues, alcohol use, depression, develop post-surgery and may take time to translate into new obesogenic behaviours which can be targeted in therapy.[14] Several studies have found that 20-30% of patients regain some weight within the first 24 months post-surgery; ironically, it is at this stage when patients may be discharged from follow up clinics.[15]

This feasibility RCT was therefore designed to determine whether ACT, delivered 15-18 months post-bariatric surgery (that is when patients are approaching discharge from secondary care), is acceptable to patients, and to determine the methods of a future, larger trial of effectiveness.

METHODS

Design

This will be a feasibility study for a single-centre, parallel group, single blind, two arm trial, with participants randomised to either 10 weeks of ACT group therapy or a Usual Care Support Group control. The trial has received a favourable ethical opinion from London-Westminster REC (18/LO/1256).

Setting

The trial will be conducted with adult (≥18 years old) patients from a London-based weight management centre.

Participants and recruitment

Inclusion criteria

Adults of any sex (aged ≥18), who have undergone any bariatric-surgery (i.e., gastric bypass, sleeve gastrectomy, gastric band and re-operations), patients more than one year out of surgery and who are willing and able to give informed consent for participation in the study.

Exclusion criteria

Suicidal ideation (score of >0 on Question 9, PHQ-9).[16] Patients who are unable to communicate in English as the intervention is delivered in English and unable to commit to attendance to 10 sessions.

Potentially eligible participants will be provided with a Participant Information Sheet (PIS) and a PHQ-9 to screen for suicidal ideation by a clinician at their regular follow-up appointment. Patients who do not attend will be posted the PIS with their routine appointment reminder letter to avoid selection-bias.

Eligible patients attending the clinic for their routine follow up appointment, who verbally express an interest in participating in the study will be approached by a researcher after their appointment and invited to read through the PIS. They will have the opportunity to ask questions about the study and, if the patient wishes to participate, an appointment will be made with the researcher to obtain written consent, and for baseline data collection and randomisation.

Randomisation

Participants who meet the eligibility criteria will be randomised to either ACT or the Usual Care Support Group. Randomisation will be conducted by a researcher, independent of the trial, running R program scripts.[17] The randomisation method uses permuted blocks of variable size, randomly varying from two to six participants. All eligibility criteria will be checked before randomisation by the researcher who will then request randomisation. Each patient will be given a unique patient study number which will be sent to the independent researcher running the R program randomisation script. The program generates a randomised patient study number assigning them to either the ACT or the Usual Care Support Group. This is emailed back to the study clinician (SS) who then informs the participant of their allocation and provides details of where and when to attend. Equal number are allocated to each condition. The flow of participants through the study is depicted in Figure 1.

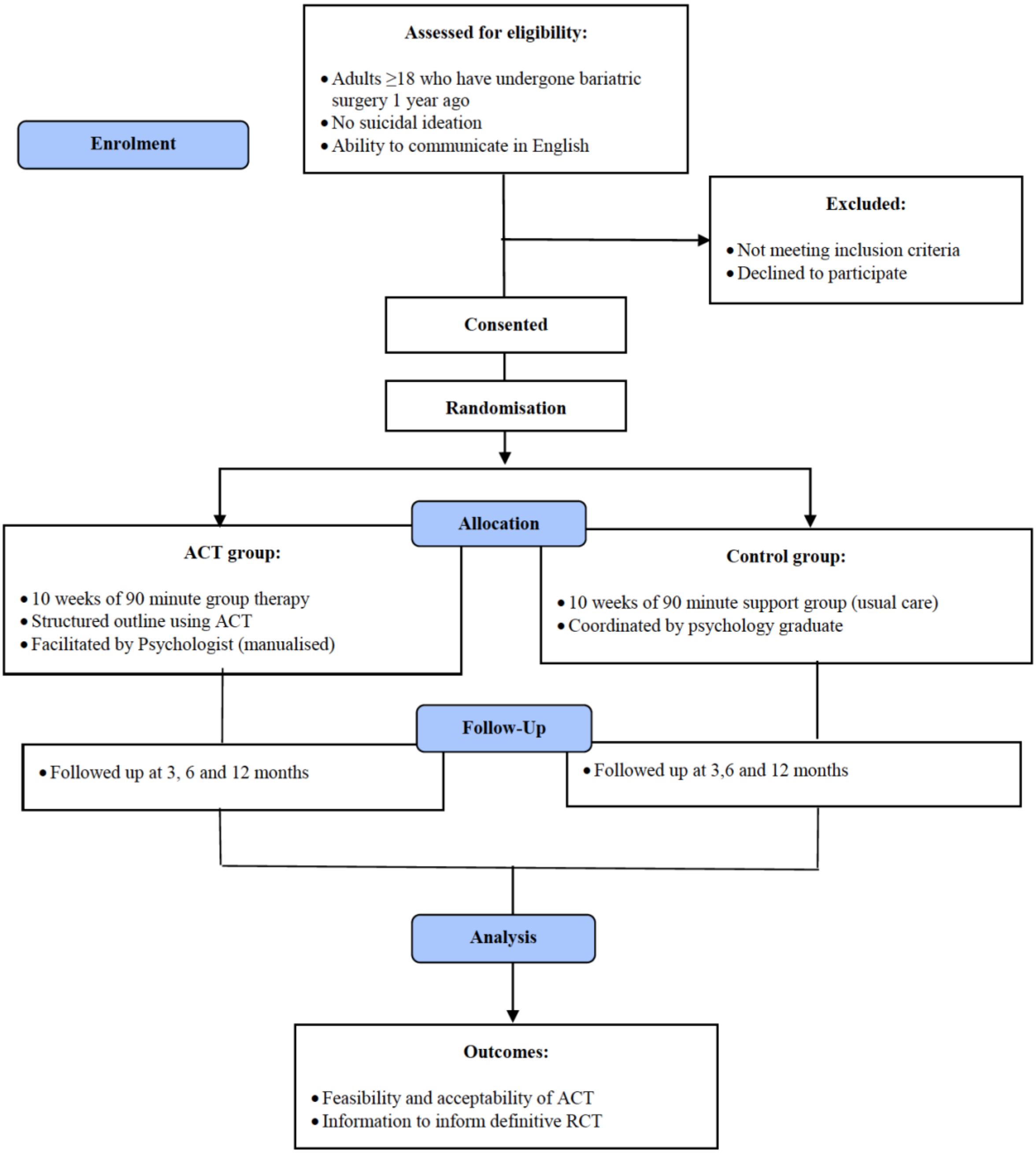

Figure 1: Participants through the study.

Blinding

In a randomised comparison of this type, patient blinding is clearly impossible. The researcher (LC) will however be blind to randomisation status and will be asked to give their opinion on randomisation status to determine whether blinding is adequate.

Sample size

As this is a feasibility study and no hypothesis testing will occur, a power calculation has not been conducted. Sample sizes between 24 and 50 have been recommended for feasibility studies,[18] however to allow for loss to follow up, estimated at 30%, we will aim to recruit 58 participants. Loss to follow up and intervention and Usual Care Support Group attrition, including reasons, will be recorded to inform power calculations for a definitive RCT. This information will be recorded in a consort diagram.[19]

Intervention

ACT group therapy

Ten 90-minute sessions will be delivered weekly by a clinical psychologist trained in bariatric psychological support and ACT. Components of the intervention are shown in Table 1. The ACT intervention has been adapted from a manual used in a previous pilot trial for bariatric surgery patients who experienced weight regain.[20] The following adaptations were made by ACT trained members of the research team (EB, DLB) and agreed with the multidisciplinary team and service user advisors: (1) Reduced emphasis on eating behaviours and increased emphasis on living in line with personal values. For instance, the necessity for daily weigh-ins, calorie counting, and food recording for all participants has been removed. Instead, participants are encouraged to set personalised, achievable goals (which may or may not involve specific eating behaviours) which move them towards their self-defined values. The manual includes support to enhance values clarity. (2) A stance of self-compassion is actively encouraged throughout the intervention.

The intervention focuses on fostering key ACT processes, including acceptance of difficult internal processes (e.g., thoughts, feelings, urges), defusion from thoughts, values clarification, willingness and committed action, self as context and linking values to present moment decision-making.[11] The aim is to enhance participants' ability to adhere to the restrictive postoperative diet in order to prevent weight regain whilst maintaining or improving quality of life.

As part of the intervention, each week participants complete two measures of ACT processes: The Bullseye (adapted from Lundgren's Bullseye Worksheet (2012))[21] which facilitates values clarification and prompts them to consider how closely they are living in line with their values, and the CompACT: a brief measure of ACT processes designed for clinical and research use.[22] The intervention also includes standard psychoeducation and behavioural interventions for weight management post-bariatric surgery. Homework is set and reviewed weekly to reinforce in-session learning. In addition, and at the therapist's discretion, participants are alerted to reinforcing resources such as ACT-relevant 'YouTube' videos.

Table 1: ACT intervention components.

Session	Psychoeducation and behavioural components	Acceptance-based components
1	Programme overview	Limitations of experiential avoidance
2	Energy balance	Acceptance as the alternative to control
	Postoperative nutrition information	Introducing willingness
3	Physical activity guidelines	Review of acceptance and willingness
		Willingness skills continued
		Values clarification
4	Challenges of obesogenic environment	Incorporating values into behaviour
	Portion control	Behavioural flexibility
5	Protein intake after surgery	The problem with mindless eating
		Mindful decision-making
6	Handling holidays, weekends and vacations	Defusion (i.e., cognitive distancing)
7	Slowing down rate of eating	Strategies to help defusion and increase willingness
	Decreasing grazing	
8	Decreasing fat intake	Defusion review
		Urge surfing
9	Preparing for the end of group	Review of major concepts
	Distinction between lapse and relapse	Responding to decreased motivation in the long term
10	Congratulations	Continued commitment
		Final review of concepts

Control

Usual care support group

The usual care support group will comprise 10 consecutive weeks of 90-minute support groups (current usual care), coordinated by a psychology graduate with no formal therapy training. The groups will be unstructured to allow patients to talk about their difficulties in a minimally facilitated way. If patients request specific advice, self-help guidance, signposting to appropriate sources of help and onward referral to specialist services will be provided. The usual care support group will be held at the same time and venue as the ACT group, but on a different day of the week.

Treatment fidelity

The psychologist delivering the ACT intervention will receive supervision from the members of the study team who have experience delivering ACT (EB) and working with bariatric patients (SS). A sample of sessions will be observed by a member of the study team trained in ACT (EB) and assessed for fidelity to the manual using a published rating scale[23] adapted for this trial. Deviations from the manual will be recorded and addressed in supervision.

Data collection and measures

A standardised battery of measures will be completed at baseline, three, six and 12 month follow up, except for weight and the King's Obesity Staging Criteria which will be collected at baseline and 12 month follow up only to allow for meaningful change. Demographic data: self-reported medical history, age, gender, work status, educational level, ethnicity, marital status, number of dependents, current or past psychological therapy and current medications, will also be collected at baseline. Baseline and 12 month follow up data will be collected face to face to coincide with routine clinic appointments; three and six month follow up data will be collected via telephone.

Primary outcome measures

The primary outcome is the feasibility of the trial methodology. This will be measured by, (1) The willingness of participants to be randomised, measured by recording the number of participants who drop out of the study after they have been randomised, (2) the number of eligible patients attending clinic, as assessed by calculating the percentage of patients who meet eligibility criteria at screening, (3) follow-up rates, assessed by the percentage of participants retained until 12 month follow-up and providing complete datasets, (4) adherence to the intervention or control, assessed by number of sessions attended, (5) reasons for attrition, (6) the time needed to collect data and (7) the acceptability of the intervention at 12 month follow-up, as assessed by a 5 point likert scale (1="Not at all", 3="somewhat", 5="Very") to evaluate how helpful participants found the treatment, their satisfaction with it, and how likely they would be to recommend it to a friend and (8) the standard deviation of weight maintenance (<5% weight gain from baseline assessment) or weight loss, in order to estimate the required sample size for a future trial (measured at study close-out).

Secondary outcome measures

Outcomes relating to (A) obesity, health and wellbeing, (B) costs, and (C) measures of ACT processes will be collected to test the appropriateness and acceptability of measurement instruments to be used as outcomes in a full trial. The validated instruments to be used are listed below.

Obesity and health and wellbeing

The King's Obesity Staging Criteria. This measures the severity of an individual's obesity based on a four-graded set of health-related domains. It can capture health problems related to obesity and health benefits after weight loss.[24]

The International Physical Activity Questionnaire (IPAQ). This measures levels of physical activity.[25]

The Brief Mediterranean Diet Questionnaire. This measures adherence to a Mediterranean diet which is recommended as part of the intervention.[26]

The Alcohol Use Disorders Identification Test (AUDIT). This measures alcohol consumption, drinking behaviours, and alcohol-related problems.[27]

The Dutch Eating Behaviour Questionnaire (DEBQ). This measures eating behaviour in terms of restraint, emotional, and external eating.[28]

The Hospital Anxiety and Depression Scale (HADS). This measures levels of anxiety and depression.[29]

Weight in kilograms.

Costs

The Client Service Receipt Inventory (CSRI). This measures resource use by participants by asking them about their health service use and social service over the last 3 month. Questions relate to healthcare use in both primary care [general practitioner (GP) and community nursing services] and secondary care as well accident and emergency (A&E) department. Participants will be asked to report the frequency and intensity of their service use.[30]

EQ-5D-5L: This measures health-related quality adjusted life years (QALY). The five attributes of this questionnaire (mobility, self-care, usual activities,

pain/discomfort/anxiety/depression) will be summarised into a single UK-derived preference-based utility score.[31]
ICECAP-A (ICEpop CAPability measure for Adults): This is a measure of capability for the general adult population for use in economic evaluation. It focuses on wellbeing defined in a broader sense than health. The measure covers attributes of wellbeing that were found to be important to adults in the UK.[32]
ACT processes
The Distress Tolerance Scale (DTS). This measures ability to tolerate distress.[33]
The Philadelphia Mindfulness Scale (PHLMS). This measures present moment awareness and acceptance.[34]
The Drexel Defusion Scale (DDS). This measures cognitive distancing or 'defusion'.[35]
The Physical Activity Acceptance Questionnaire (PAAQ). This measures the extent to which an individual is able tolerate physical or psychological discomfort.[36]
The Food Acceptance and Awareness Questionnaire (FAAQ). This measures acceptance of food-related thoughts and urges.[37]

Tertiary qualitative outcomes

A nested qualitative, semi-structured interview study will be conducted with 12 participants (six ACT; six control) following the 12 month follow-up in order to understand the 'what, why and how come' of participants' beliefs and behaviours in order to inform the design and delivery of a future full RCT. A topic guide will prompt participants to discuss their experience of participation in the study and in the groups. The topic guide will be informed by the study and intervention processes, observations made by the study team, study psychologist, Usual Care Support Croup co-ordinator and participants during the groups. It will be developed with service user advisors and will take approximately 1 hour to complete. Interviews will be recorded and transcribed verbatim.

Statistical data analysis

Analyses will be undertaken using R program.[17] All significance tests will be 2-tailed and alpha will be set at $p<0.05$. The distribution of the study population will be characterised using descriptive summary statistic parameters, that is median, range, mean, and standard deviation. Then, we propose to use ANCOVA to calculate variations within primary and secondary outcome measures pre-treatment and at follow-up. Anonymised data from those who decline to participate will also be reported. Complete case analysis and intent-to-treat analyses (using last measurement carried forward for participants who provided baseline assessment measurements) will be conducted.
A cost-consequence analysis (CCA) will be carried out from a National Health Service (NHS) perspective. This involves listing the costs and outcomes associated with ACT and the Usual Care Support Group separately, allowing decision-makers to compare the relative value of approaches, and by calculating resource use. The intervention costs will be calculated based on the activities involved in delivering the programme such as staff salaries, administrative and managerial costs.
Total costs of the individuals in the two groups will be compared and estimated by multiplying the resource use with the unit costs of health and social care.[38] We will present the additional costs associated with the intervention and present the costs alongside the benefits (if any) identified in the trial. Sensitivity analyses to model uncertainties around all key cost and outcome parameters, and plausible ranges will be specified using information from the trial and from the literature specific to the population in the study will be conducted.

Qualitative analysis

Interview data will be analysis according to the principles of inductive thematic analysis.[39] Data will be coded and themed by at least two authors who will independently read the transcripts to identify themes. The two authors will then agree themes, which will then be further confirmed through discussion within the whole team including service user advisors.

DISCUSSION

This study aims to establish the feasibility of conducting a larger scale RCT to examine the impact of ACT on bariatric patients 15-18 months post-surgery. Previous small-scale studies of acceptance-based behavioural intervention for post-operative populations have found benefits in favour of ACT compared with usual care.[11,12,40] The different studies focused on different outcomes, but benefits identified included reduced cravings and sweet consumption, reduced disordered eating and improved body dissatisfaction and quality of life, as well as weight loss (a clinically significant average of 6 kg or 5% weight loss in treatment completers, n=23).[11,12,40] Enhanced psychological flexibility, a core ACT process, was identified as one mediator for benefits in one trial, though other processes have also been proposed as important.[9] This study will therefore examine a wide range of potential treatment outcomes and will test for possible mediators and moderators of effect.[12] In the earlier studies, the time since surgery varied widely between participants.[11,12,40] The current study will focus on patients who have reached a critical stage in their treatment; a stage which has been identified as high risk for weight regain and at which point they are currently discharged from services.

Despite the potential benefit of psychological intervention and recommendations within various guidelines that it should be offered to bariatric patients, psychological support is not provided as part of routine post-operative care.[7] Such support could potentially reduce the high costs to the patient and to society of weight regain, re-operations and the return of physical and psychological co-morbidities post bariatric surgery.[7] This feasibility trial is the first step in helping to determine whether ACT group therapy is acceptable to post-bariatric surgery patients, and will provide insight into the optimal methods to employ for a future randomised controlled trial to determine its effectiveness.

ACKNOWLEDGEMENTS
This paper presents independent research funded by the National Institute for Health Research (NIHR) under its Research for Patient Benefit (RfPB) Programme (Grant Reference Number PB-PG-0816-20012). The views expressed are those of the author(s) and not necessarily those of the NHS, the NIHR or the Department of Health and Social Care. Non-financial support has been provided by Noclor research support service.
Funding: Funded by the National Institute for Health Research (NIHR) under its Research for Patient Benefit (RfPB) Programme (Grant Reference Number PB-PG-0816-20012)
Conflict of interest: None declared
Ethical approval: This trial received favourable opinion by London – Westminster REC and the Health Research Authority

REFERENCES
1. World Health Organization. Obesity and Overweight, 2018. Available at: https://www.who. int/news-room/fact-sheets/detail/obesity-and-overweight. Accessed 14 June 2019.
2. NHS Digital. Statistics on obesity, physical activity and diet, 2018. Available at: https://digital.nhs. uk/data-and-information/publications/statistical/ statistics-on-obesity-physical-activity-and-diet/ statistics-on-obesity-physical-activity-and-diet-england-2018. Accessed 14 June 2019.
3. Courcoulas AP, King WC, Belle SH, Berk P, Flum DR, Garcia L, et al. Seven-year weight trajectories and health outcomes in the longitudinal assessment of bariatric surgery (LABS) study. JAMA Surg. 2018;153(5):427-34.
4. Cooper TC, Simmons EB, Webb K, Burns JL, Kushner RF. Trends in weight regain following roux-en-Y gastric bypass (RYGB) bariatric surgery. Obesity Surg. 2015;25(8):1474-81.
5. Ostlund MP, Backman O, Marsk R, Stockeld D, Lagergren J, Rasmussen F, et al. Increased admission for alcohol dependence after gastric bypass surgery compared with restrictive bariatric surgery. JAMA Surg. 2013;148(4):374-7.
6. Scholtz S, Goldstone AP, le Roux CW. Changes in reward after gastric bypass: The advantages and disadvantages. Curr Atherosclerosis Reports. 2015;17(10):61.
7. National Institute for Health and Care Excellence. Obesity: Identification, assessment and management, 2014. Available at: https://www.nice. org.uk/guidance/cg189. Accessed 14 June 2019.
8. Stewart F, Avenell A. Behavioural interventions for severe obesity before and/or after bariatric surgery: A systematic review and meta-analysis. Obesity Surg. 2016;26(6):1203-14.
9. Marek RJ, Ben-Porath YS, Dulmen MHMV, Ashton K, Heinberg LJ. Using the presurgical psychological evaluation to predict 5-year weight loss outcomes in bariatric surgery patients. Surg Obes Relat Dis. 2017;13(3):514-21.
10. O'Reilly GA, Cook L, Spruijt-Metz D, Black DS. Mindfulness-based interventions for obesity-related eating behaviours: A literature review. Obesity Reviews: An Official J Int Assoc Study Obes. 2014;15(6):453-61.
11. Forman E, Butryn ML, Hoffman KL, Herbert JD. An open trial of an acceptance-based behavioral intervention for weight loss. Cogn Behav Prac. 2009;16(2):223-35.
12. Weineland S, Arvidsson D, Kakoulidis TP, Dahl J. Acceptance and commitment therapy for bariatric surgery patients, a pilot RCT. Obesity Res Clin Prac. 2012;6(1):e1-e90.
13. Ogden J, Hollywood A, Pring C. The impact of psychological support on weight loss post weight loss surgery: A randomised control trial. Obesity Surg. 2015;25(3):500-5.
14. Odom J, Zalesin KC, Washington TL, Miller WW, Hakmeh B, Zaremba DL, et al. Behavioral predictors of weight regain after bariatric surgery. Obesity Surg. 2010;20(3):349-56.
15. Ferchak CV, Meneghini LF. Obesity, bariatric surgery and type 2 diabetes – a systematic review. Diabetes/Metab Res Rev. 2004;20(6):438-45.
16. Kroenke K, Spitzer RL, Williams JB. The PHQ-9: Validity of a brief depression severity measure. J Gen Intern Med. 2001;16(9):606-13.
17. R Core Team. R: A language and environment for statistical computing. R Foundation for Statistical Computing 2018. Vienna, Austria. Available at: https://www.R-project.org/. Accessed 14 June 2019.

18. Lancaster GA, Dodd S, Williamson PR. Design and analysis of pilot studies: Recommendations for good practice. J Eva Clin Prac. 2004;10(2):307-12.
19. Moher D, Schulz KF, Altman DG. The CONSORT statement: Revised recommendations for improving the quality of reports of parallel-group randomised trials. Lancet (London, England) 2001;357(9263):1191-4.
20. Bradley LE, Forman EM, Kerrigan SG, Butryn ML, Herbert JD, Sarwer DB. A pilot study of an acceptance-based behavioral intervention for weight regain after bariatric surgery. Obesity Surg. 2016;26(10):2433-41.
21. Lundgren T, Louma J, Dahl, J, Strohsal K, Melin L. The bull's-eye values survey: A psychometric evaluation. Cogn Behav Prac. 2012;19(4):518-26.
22. Francis AW, Dawson DL, Golijani-Moghaddam N. The development and validation of the Comprehensive assessment of Acceptance and Commitment Therapy processes (CompACT). J Contextual Behav Sci. 2016;5(3):134-45.
23. Gifford EV, Kohlenberg BS, Hayes SC, Antonuccio DO, Piasecki MM, Rasmussen-Hall ML, et al. Acceptance-based treatment for smoking cessation. BehavTherap 2004;35:689-705.
24. Sharma AM, Kushner RF. A proposed clinical staging system for obesity. Int J Obes. 2009;33:289-95.
25. Hallal P, Victora C. Reliability and validity of the international physical activity questionnaire (IPAQ). Med Sci Sports Exercise. 2004;36(3):556.
26. Martinez-Gonzalez MA, Garcia-Arellano A, Toledo E, Salas-Salvado J, Buil-Cosiales P, et al. A 14-Item Mediterranean Diet Assessment Tool and Obesity Indexes among High-Risk Subjects: The PREDIMED Trial. PLoS ONE. 2012;7(8):e43134.
27. Saunders JB, Aasland OG, Babor TF, de la Fuente JR, Grant M. Development of the Alcohol Use Disorders Identification Test (AUDIT): w collaborative project on early detection of persons with harmful alcohol consumption. II. Addiction. 1993;8:791-804.
28. Van Strien T, Frijters J, Bergers G, Defares PB. The Dutch Eating Behavior Questionnaire (DEBQ) for assessment of restrained, emotional, and external eating behaviour. Int J Eating Disord. 1986;5(2):295-315.
29. Zigmond AS, Snaith RP. The Hospital Anxiety and Depression Scale. Acta Psychiatr Scand. 1983;67(6):361-70.
30. Beecham J, Knapp M. Costing psychiatric interventions. In: Thornicroft G (ed). Measuring Mental Health Needs. 2nd edition. Gaskell, London, UK; 2001: 200-224.
31. The EuroQol Group. EuroQol-a new facility for the measurement of health-related quality of life. Health Policy. 1990;16(3):199-208.
32. Al-Janabi H, Flynn T, Coast J. Development of a self-report measure of capability wellbeing for adults: the ICECAP-A. Quality Life Res. 2012;21:167-76.
33. Simons JS, Gaher RM. The distress tolerance scale: Development and validation of a self-report measure. Motivat Emot. 2005;29:83-102.
34. Cardaciotto L, Herbert JD, Forman EM, Moitra E, Farrow V. The assessment of present-moment awareness and acceptance: The Philadelphia mindfulness scale. Assessment. 2008;15:204-23.
35. Forman EM, Herbert JD, Juarascio AS, Yeomans PD, Zebell JA, Goetter EM, et al. The Drexel defusion scale: A new measure of experiential distancing. J Context Behav Sci. 2012;1:55-65.
36. Butryn ML, Arigo DR, Raggio GA, Kaufman AI, Kerrigan SG, Forman EM. Measuring the Ability to Tolerate Activity-Related Discomfort: Initial Validation of the Physical Activity Acceptance Questionnaire (PAAQ). J Phys Act Health. 2015;12(5):717-6.
37. Juarascio A, Forman E, Timko CA, Butryn M, Goodwin C. The development and validation of the food craving acceptance questionnaire. Eating Behaviors. 2011;12:182-7.
38. Curtis L, Burns A. Unit Costs of Health and Social Care 2018. Personal Social Services Research Unit, University of Kent, Canterbury. 2018.
39. Braun V, Clarke V. Using thematic analysis in psychology. Qual Res Psychol. 2006;3(2):77-101.
40. Bradley LE, Forman EM, Kerrigan SG, Goldstein SP, Butryn ML, Thomas JG, et al. Project HELP: A remotely delivered behavioral intervention for weight regain after bariatric surgery. Obesity Surg. 2017;27(3):586-98.
