# Supplementary material for: Addressing a critical need: A randomised controlled feasibility trial of acceptance and commitment therapy for bariatric surgery patients at 15–18 months post-surgery
Source: PLoS One. 2023 Apr 25;18(4):e0282849. doi: 10.1371/journal.pone.0282849 (PMC10128967; doi:10.1371/journal.pone.0282849)
Supplement: Attachment — Submitted filename: Response to Reviewers.docx [file pone.0282849.s011.docx]

Katrien Janin
Staff Editor
PLOS ONE

Dear Dr Janin

Thank you for your email. We have now revised the manuscript in accordance with the new minor points that Reviewer 2 has made in their second review. The changes are listed below.

We hope that the manuscript is now acceptable for publication.

Reviewer #1: (No Response)

Author response: we thank Reviewer 1 for their positive review.

Reviewer #2: The authors have done a reasonable job of responding to my earlier comments.
The qualitative analysis is still a little thin, but I think that is the nature of the data collected and is unlikely to improve.

Author response: we thank Reviewer 2 for their very detailed reviews. We agree with the above comment.

Reviewer 2: Some further minor edits are suggested prior to publication
The following sentence does not make sense: Patients who expressed suicidal ideation (score >0 on 125 Q9, PHQ-9) or at psychiatry assessment [26], were unable to communicate in English or to commit to 126 attendance were excluded.

Author Response: Thank you, we have restructured the sentence accordingly: “Patients who expressed suicidal ideation (score >0 on Q9 of the PHQ-9 [26] or at psychiatry assessment), or who were unable to communicate in English, or who were unable to commit to attendance were excluded.”

Reviewer 2: the cost consequence method is not well described. What was included in the analysis? How was this done?

Author Response: The requested detail has now been added with references (p11)

Reviewer 2: It is not clear how the ACT group was associated with a 0.07 QALY reduction in mean incremental QALY if mean QALYs accrued from baseline to 12 month 346 follow-up were: ACT (n=13) = 0.61 SD 0.26; SGC (n=19) = 0.59 SD 0.37.

Author Response: this as now been clarified (p18-19)

Reviewer 2: The authors comment that data collection was feasible and acceptable, but less than 50% completed 12 month data collection. Tis suggests problems with data collection and trial retention.

Author Response: it is unclear to where in the manuscript the reviewer is referring, we have been clear about trial retention issues throughout, we have now adapted the following existing sentence (p25) to emphasise the point: “It appeared that although data collection was hampered by lack of retention, other trial processes (i.e. recruitment, consent, data collection in those retained in the trial) would be feasible for a future trial but that the intervention, as delivered, may not be.”

Editor comment: Any changes to the reference list should be mentioned in the rebuttal letter that accompanies your revised manuscript. If you need to cite a retracted article, indicate the article’s retracted status in the References list and also include a citation and full reference for the retraction notice.

Author Response: 3 references have now been added to support the additional information requested about the cost-consequence analysis:

44. National Institute for Health and Care Excellence. Medical technologies evaluation programme methods guide 2017. Available at: <https://www.nice.org.uk/process/pmg33/chapter/evidence-synthesis-and-cost-consequence-analysis>. Accessed 9 Feb 2023.
45. Drummond M. Methods for the economic evaluation of Health Care Programmes. Oxford, United Kingdom: Oxford University Press; 2015.
46. Curtis LA, Burns A. Unit Costs of Health and Social Care 2018 [Internet]. University of Kent; 2018. Available from: https://kar.kent.ac.uk/id/eprint/70995

Thank you for your consideration, we look forward to your response.

kind regards

Elizabeth Barley PhD CPsychol AFBPsS RGN PGCAP FHEA
Professor of Mental Health Sciences and Nursing

School of Health Sciences

University of Surrey

30 Priestley Road

Surrey Research Park

Guildford

Surrey

GU2 7YH
